# Prognostic Biomarkers in Breast Cancer via Multi-Omics Clustering Analysis

**DOI:** 10.3390/ijms26051943

**Published:** 2025-02-24

**Authors:** Federica Malighetti, Matteo Villa, Alberto Maria Villa, Sara Pelucchi, Andrea Aroldi, Diego Luigi Cortinovis, Stefania Canova, Serena Capici, Marina Elena Cazzaniga, Luca Mologni, Daniele Ramazzotti, Nicoletta Cordani

**Affiliations:** 1 Department of Medicine and Surgery, University of Milano-Bicocca, 20900 Monza, Italy; f.malighetti@campus.unimib.it (F.M.); m.villa96@campus.unimib.it (M.V.); a.villa133@campus.unimib.it (A.M.V.); sara.pelucchi@unimib.it (S.P.); andrea.aroldi@unimib.it (A.A.); diego.cortinovis@unimib.it (D.L.C.); marina.cazzaniga@unimib.it (M.E.C.); luca.mologni@unimib.it (L.M.); daniele.ramazzotti@unimib.it (D.R.); 2 Oncology Unit, Fondazione IRCCS San Gerardo dei Tintori, 20900 Monza, Italy; stefania.canova@irccs-sangerardo.it; 3 Phase 1 Unit, Fondazione IRCCS San Gerardo dei Tintori, 20900 Monza, Italy; serena.capici@irccs-sangerardo.it

**Keywords:** breast cancer, multi-omics landscape, prognostic biomarkers

## Abstract

Breast cancer (BC) is a highly heterogeneous disease with diverse molecular subtypes, which complicates prognosis and treatment. In this study, we performed a multi-omics clustering analysis using the Cancer Integration via MultIkernel LeaRning (CIMLR) method on a large BC dataset from The Cancer Genome Atlas (TCGA) to identify key prognostic biomarkers. We identified three genes—*LMO1*, *PRAME*, and *RSPO2*—that were significantly associated with poor prognosis in both the TCGA dataset and an additional dataset comprising 146 metastatic BC patients. Patients’ stratification based on the expression of these three genes revealed distinct subtypes with markedly different overall survival (OS) outcomes. Further validation using almost 2000 BC patients’ data from the METABRIC dataset and RNA sequencing data from therapy-resistant cell lines confirmed the upregulation of *LMO1* and *PRAME*, respectively, in patients with worse prognosis and in resistant cells, also suggesting their potential role in drug resistance. Our findings highlight *LMO1* and *PRAME* as potential biomarkers for identifying high-risk BC patients and informing targeted treatment strategies. This study provides valuable insights into the multi-omics landscape of BC and underscores the importance of personalized therapeutic approaches based on molecular profiles.

## 1. Introduction

Cancer is a highly complex disease characterized by molecular, cellular, and environmental interactions [1]. This heterogeneity is reflected in genetic, epigenetic, and phenotypic diversity [2], all of which contribute to different treatment responses, even among patients with similar diagnoses [3]. Such heterogeneity manifests in the coexistence of several cell subtypes that dynamically adapt to their microenvironment. Clonal evolution of tumor cells can enable immune evasion and resistance to therapeutic interventions [4].

Breast cancer (BC) is the most common cancer worldwide and represents a very heterogeneous disease [2]. It is the second leading cause of cancer-related deaths among women, following lung cancer. BC is clinically classified into hormone receptor (HR)-positive, HER2-positive (HER2+), and triple-negative breast cancer (TNBC) subtypes based on the expression of estrogen receptors (ER), progesterone receptors (PR), and HER2 receptors. These molecular profiles guide treatment decisions, with tailored therapies targeting specific receptors or addressing receptor absence in TNBC. Early detection improves outcomes significantly, with a 99% five-year relative survival rate for localized cases. HR+/HER2-negative is the most common subtype, accounting for over 75% of BC cases [5]. TNBC, representing 10–20% of BC cases [6], is highly aggressive, with a peak relapse within three years from diagnosis and predominant hepatic, pulmonary, and central nervous system metastases [7].

Precision medicine addresses BC heterogeneity by utilizing PAM50 subtypes and risk of recurrence (ROR) scores to classify intrinsic molecular subtypes (Luminal A, Luminal B, HER2-enriched, Basal-like, or Normal-like) or recurrence risks [8,9].

The use of advanced high-throughput experimental technologies is becoming increasingly prevalent in generating extensive omics datasets, including those derived from genomics and transcriptomics, across diverse scientific fields. These datasets frequently originate from the same patients, allowing for a comprehensive examination of both molecular and clinical characteristics. The integration of these datasets has significantly advanced efforts to identify and categorize molecular subtypes of cancer, which are closely associated with patient outcomes. Consequently, characterizing these subtypes has emerged as a critical area of focus in oncology research, potentially offering insights that could inform personalized therapeutic strategies [10].

In this study, we aimed to uncover biologically relevant subtypes of BC using multi-omics data to improve our understanding of tumor heterogeneity and its implications for prognosis. To this end, we analyzed BC data from the latest multi-omics dataset released by The Cancer Genome Atlas (TCGA) [11,12], encompassing nearly 1000 primary tumors, and from a validation cohort of 146 metastatic BC cases [13]. We performed a standardized pipeline for raw data preprocessing and employed the Cancer Integration via MultIkernel LeaRning (CIMLR) clustering method to identify subtypes within these multi-omics datasets, particularly focusing on the molecular characteristics significantly linked to prognosis [14].

Multi-omics clustering analysis enabled the identification of molecularly distinct subgroups that may respond differently to treatment and exhibit varying survival outcomes. We then identified genes differentially expressed among these clusters, determining a set of candidate molecular features. To determine their prognostic relevance, we performed survival analysis using regularized Cox regression, ultimately identifying potential biomarkers associated with patient outcomes (Supplementary Figure S1). Our findings were further validated using independent datasets, including almost 2000 BC patients’ data from the METABRIC project and resistant cell lines [15], reinforcing their potential clinical utility.

Our analysis identified *LMO1* and *PRAME* as promising prognostic biomarkers in BC patients, thereby validating our framework as a robust, generalizable approach for discovering prognostic biomarkers in cancer.

## 2. Results

### 2.1. Biomarkers Identification

We conducted multi-omics clustering analysis using CIMLR [14] on the latest dataset from TCGA [11,12], which includes 985 BC patients with multi-omics data. This analysis identified clusters representing distinct cancer subtypes. These clusters were further analyzed to detect differentially expressed genes, resulting in a preliminary set of differentially expressed genes (Supplementary Data S1), which characterized the identified subtypes.

To assess the prognostic relevance of these genes, we performed regularized Cox regression analysis, identifying 32 significant genes (Supplementary Data S1). Through refinement and validation using the existing literature, we categorized these genes as oncogenes or tumor suppressors, confirming whether poorer prognosis was associated with higher oncogene expression or lower tumor suppressor expression. This process reduced the list to 20 significant genes (Supplementary Table S1), which were also consistent with the literature.

For additional validation, we analyzed a second dataset comprising 146 metastatic BC cases [13] and performed regularized regression analysis using the 20 identified genes. From this analysis, three genes—*LMO1*, *PRAME*, and *RSPO2*—emerged as highly significant prognostic biomarkers in both cohorts.

### 2.2. Clustering Analysis

**Figure 1:**
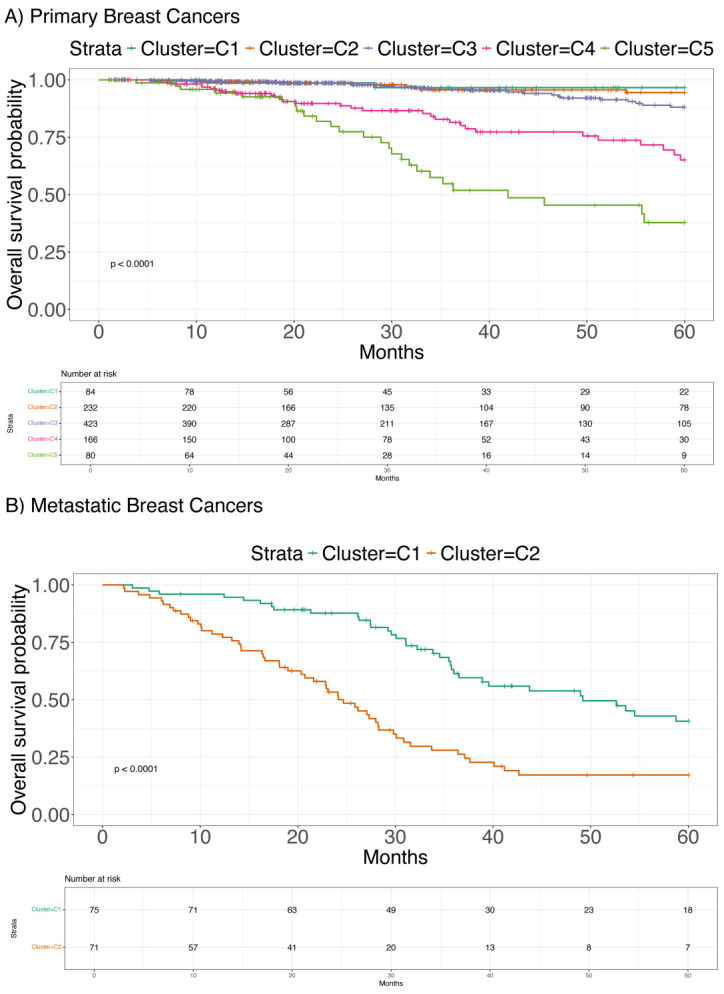
Kaplan–Meier survival analysis showing overall survival (OS) differences among subtypes identified via hierarchical clustering based on the three selected genes (*LMO1*, *PRAME*, and *RSPO2*). (**A**) shows the five subtypes detected in the TCGA primary breast cancer dataset (Cancer Genome Atlas 2012; Blum, Wang et al., 2018) [11,12] with significantly different OS (*p* < 0.001). (**B**) illustrates the two subtypes detected in the metastatic breast cancer dataset (Pleasance, Titmuss et al., 2020) [13], also with significantly different OS (*p* < 0.001).

We performed a clustering analysis considering the three genes selected by the regularized regression analysis. This was performed by computing a risk score for each patient based on the coefficients estimated by regularized regression and then stratifying the patients based on these risk scores using hierarchical clustering. This analysis revealed five distinct subtypes with significantly different overall survival (OS) (*p* < 0.001) in the TCGA primary BC dataset (Figure 1A). Similarly, two subtypes with significantly different OS (*p* < 0.001) were identified in the metastatic BC dataset [13] (Figure 1B). In both cases, the three selected genes (*LMO1*, *PRAME*, and *RSPO2*) were differentially expressed.

**Figure 2:**
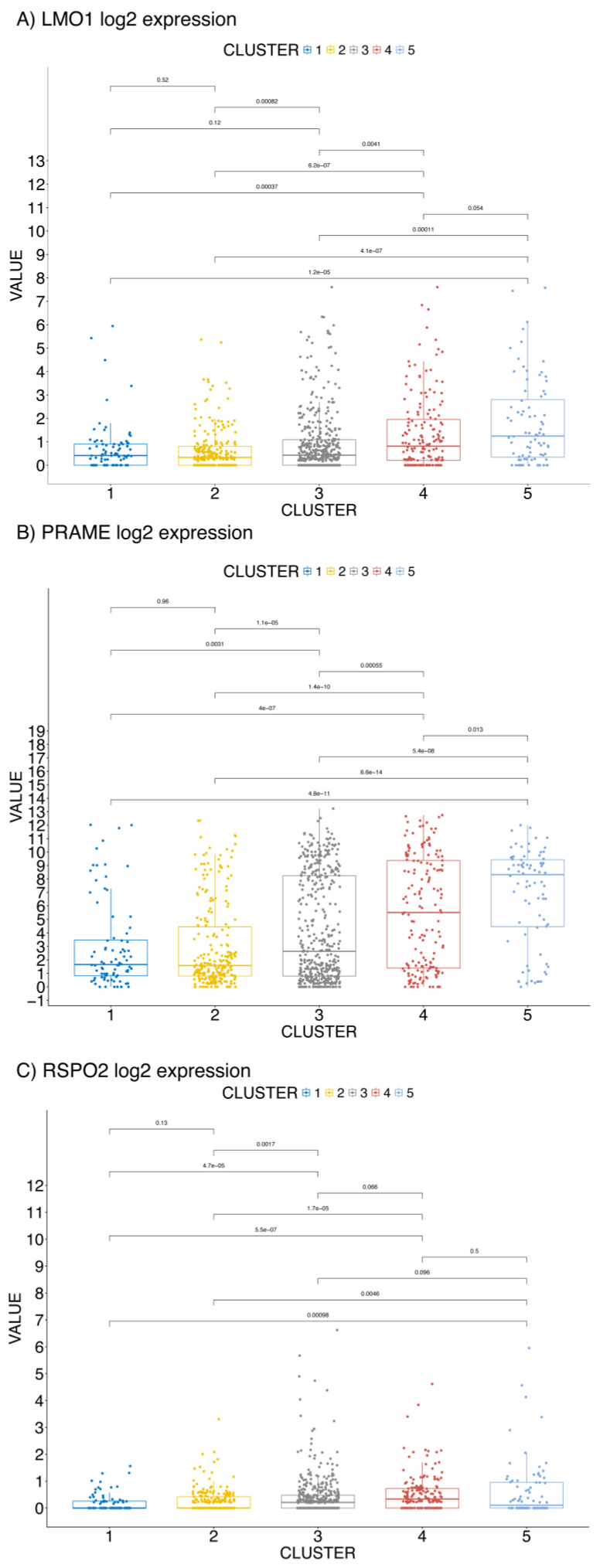
The expression profile (Log2 expression value) of *LMO1* (**A**), *PRAME* (**B**), and *RSPO2* (**C**) in the clusters from TCGA 985 BC [11,12].

The transcriptomic profile of LMO1 in the TCGA cohort is markedly elevated in Cluster 5 (Figure 2A), which also shows the worst prognosis (Figure 1A). This finding aligns with the existing literature, which identifies *LMO1* as an oncogene implicated in T-cell Acute Lymphoblastic Leukemia (T-ALL) [16] and neuroblastoma [17]. Conversely, *LMO4*, another LIM domain protein, is broadly expressed in human tissues, including over 50% of breast tumors, where it reduces the differentiation of breast cancer epithelial cells and functions as an oncogene [18].

Similarly, gene expression of the tumor antigen Preferentially Expressed Antigen of Melanoma (PRAME), a repressor of the retinoic acid receptor [19], is significantly elevated in the TCGA Cluster 5 (Figure 2B), consistent with previous reports linking high PRAME levels to worse prognoses [20].

Finally, we observed overexpression of *RSPO2* mRNA in the TCGA Cluster 5 (Figure 2C). RSPO2 is a secreted glycoprotein that stimulates Wnt/β-catenin signaling and functions as a cancer driver [21,22,23].

Notably, the three genes were also found to be correlated with the TCGA’s annotated PAM50 subtypes, as they were significantly overexpressed in the more aggressive basal subtype, including triple-negative breast cancer (TNBC) (Supplementary Figures S2–S4). However, the two stratifications are not equivalent (Supplementary Figure S5); thus, our analysis may capture additional molecular differences beyond the PAM50 subtype classification. This finding corroborates our results, further emphasizing the prognostic relevance of these genes.

**Figure 3:**
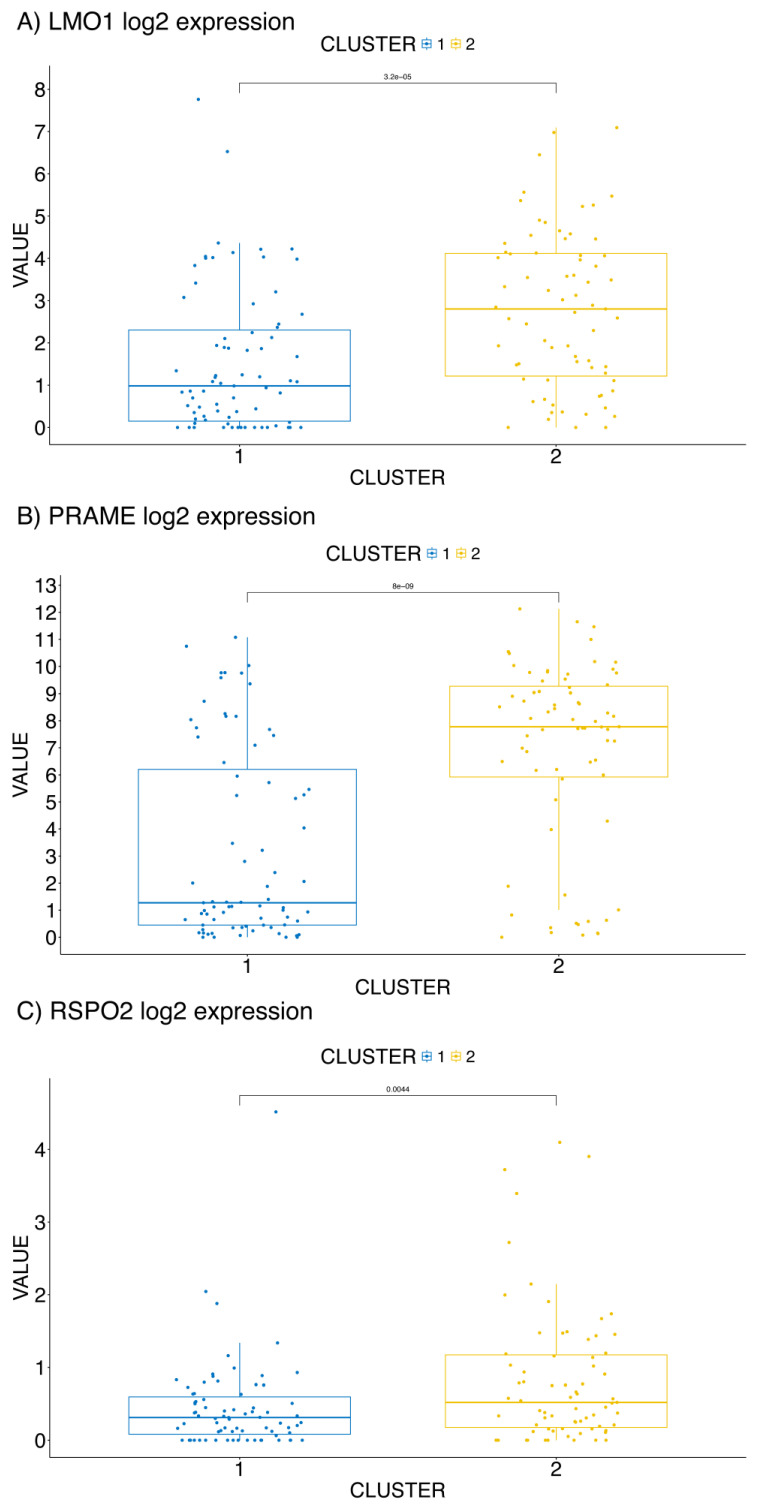
The expression profile (Log2 expression value) of *LMO1* (**A**), *PRAME* (**B**), and *RSPO2* (**C**) in the clusters from the 146 metastatic BC [13].

To further validate the prognostic significance of *LMO1*, *PRAME*, and *RSPO2*, we also verified the expression levels of the three genes in the metastatic BC dataset [13], confirming that *LMO1*, *PRAME*, and *RSPO2* were consistently overexpressed in Cluster 2 showing worst survival (Figure 3), reinforcing their roles as key prognostic biomarkers.

### 2.3. Validation on the METABRIC Dataset

**Figure 4:**
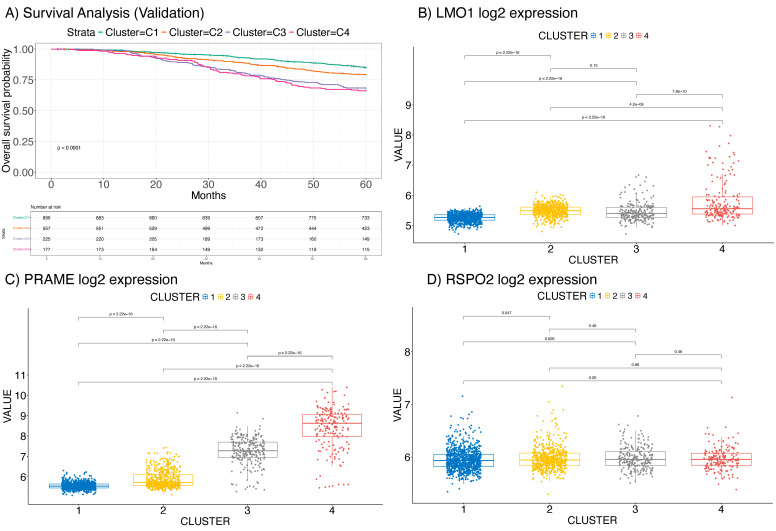
Kaplan–Meier survival analysis showing overall survival (OS) differences among sub-types identified via clustering based on the three selected genes (*LMO1*, *PRAME*, and *RSPO2*), also with significantly different OS (*p* < 0.001) (**A**) depicts four subtypes in the METABRIC primary breast cancer dataset. The expression profile (Log2 expression value) of *LMO1* (**B**), *PRAME* (**C**), and *RSPO2* (**D**) in the clusters from the 1980 patients affected by BC from METABRIC.

To further validate the three identified genes (*LMO1*, *PRAME*, and *RSPO2*) as prognostic biomarkers in an external cohort, we considered a dataset comprising 1980 patients from the METABRIC database [24,25,26], from which the PAM50 model was delineated. Clustering analysis again revealed five distinct subtypes with significantly different overall survival (OS) (*p* < 0.001) in this dataset of BC (Figure 4A). The findings indicated a robust association of *LMO1* and *PRAME* with patient prognosis (Figure 4B,C), while *RSPO2* demonstrated a non-significant association (Figure 4D).

### 2.4. Expression of the Identified Biomarkers on Resistant Cell Lines and Metastatic BC Samples

**Figure 5:**
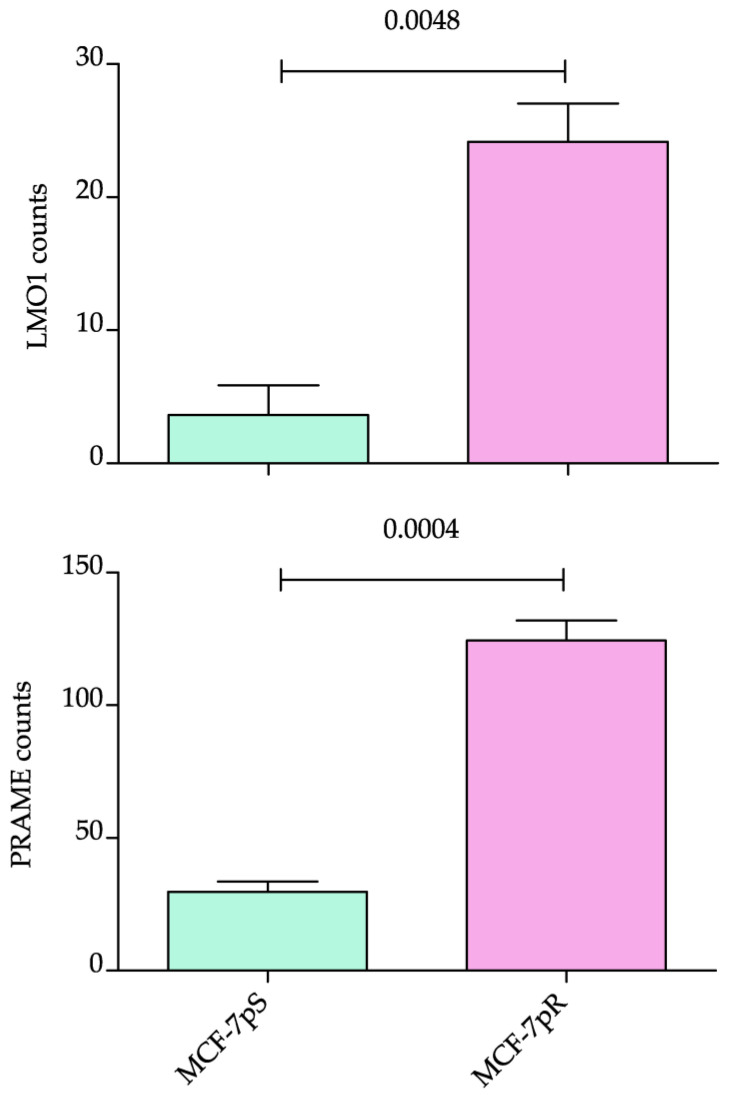
Overexpression of LMO1 and PRAME in RNA-seq normalized counts data. Values represent the mean of three internal replicates [15].

Genes whose expression is positively associated with an aggressive phenotype and poor outcome may also be drivers of resistance. Thus, we verified the expression levels of *LMO1* and *PRAME* in the RNAseq data generated in the study by Cordani and colleagues [15]. Both genes were identified as differentially expressed in RNAseq data comparing MCF-7 cells to MCF-7 cells resistant to palbociclib [15]. Notably, *LMO1* and *PRAME* were strongly upregulated in the resistant cells, further highlighting their strong association with aggressive breast cancer phenotypes. These findings underscore the potential role of these genes in mediating resistance to therapy and their relevance as biomarkers for identifying high-risk and treatment-resistant cases (Figure 5). In addition, we performed RT-qPCR to validate the three targets and confirm the data observed in the RNA-seq experiments. Moreover, we confirmed that all three candidate biomarkers are upregulated also in triple-negative breast cancer cell lines (see Supplementary Figure S6).

Finally, we analyzed an independent dataset from The Metastatic Breast Cancer Project (https://mbcproject.org/) consisting of 150 breast cancer tumors, including 59 from patients without metastasis and 91 from patients with metastasis. This analysis revealed significantly higher expression of *PRAME* and *RSPO2* in metastatic tumors, further supporting their potential role in disease progression. While *LMO1* expression was also elevated in metastases, the difference was not statistically significant. These findings provide additional insight into the clinical relevance of our identified biomarkers and are included in Supplementary Data S2.

## 3. Discussion

This study identified and validated potential prognostic biomarkers in BC by leveraging the latest multi-omics dataset from TCGA. Through multi-omics clustering and survival association analysis, we identified a set of differentially expressed genes significantly linked with BC’s prognosis. Our findings highlight three genes—*LMO1*, *PRAME*, and *RSPO2*—as candidate prognostic biomarkers.

The clustering analysis, performed on both primary and metastatic BC datasets, revealed distinct BC subtypes with significantly different OS, confirming the molecular heterogeneity of this cancer. The identification of five subtypes in the primary BC cohort and two subtypes in metastatic BC, based on the expression of *LMO1*, *PRAME*, and *RSPO2*, underscores the importance of these genes in potentially shaping the clinical outcome of patients. Specifically, Cluster 5 in the primary BC cohort and Cluster 2 in the metastatic cohort were strongly associated with the worst prognosis, aligning with the elevated expression levels of the three genes. These results are consistent with the existing literature that links these genes to cancer progression, aggressiveness, and poor prognosis.

Among the three biomarkers, *LMO1* stands out as a key oncogene that is overexpressed in BC subtypes with the worst prognosis. Previous studies have shown that *LMO1* is involved in T-cell Acute Lymphoblastic Leukemia (T-ALL) and neuroblastoma, and its role in BC, particularly in the TCGA Cluster 5, supports its potential as a marker for aggressive cancer phenotypes. Likewise, *PRAME*, a known repressor of the retinoic acid receptor, is implicated in promoting cancer cell growth and resistance to therapy. In our study, its elevated expression in the TCGA Cluster 5 of primary BCs and in Cluster 2 metastatic BC cohorts reinforces its relevance as a prognostic factor. *RSPO2*, which stimulates Wnt/β-catenin signaling, is another key contributor to tumor progression and metastasis. Our findings of increased *RSPO2* expression in the worst-prognosis clusters further support its role as a cancer driver, particularly in BC subtypes with aggressive phenotypes.

Furthermore, these biomarkers were also associated with basal-like tumors from the PAM50 subtyping system, known for its aggressive behavior and poor therapeutic response. This observation further highlights the clinical significance of these genes, particularly in identifying high-risk patients who may benefit from more aggressive treatment strategies.

Additionally, our validation on both patients’ data from the METABRIC project and on cell lines, particularly those resistant to the drug palbociclib, demonstrated that *LMO1* and *PRAME* were strongly upregulated in both patients with worse prognosis and in resistant MCF-7 cells, suggesting that these genes may also play a role in mediating resistance to therapy. The strong association of these genes with both aggressive BC phenotypes and drug resistance underscores their potential as biomarkers for identifying patients at high risk for poor prognosis and treatment resistance.

In conclusion, this study identifies *LMO1* and *PRAME* as highly significant biomarkers that can improve our understanding of BC prognosis, particularly in aggressive subtypes such as TNBC. These findings suggest that these biomarkers may not only help identify patients with poor outcomes but could also inform the development of targeted therapies to overcome drug resistance. Future studies should explore the mechanisms by which these genes contribute to BC progression and resistance, as well as the potential for incorporating them into clinical practice for personalized treatment strategies.

This study has limitations. First, further investigation is needed to understand the mechanisms of action and the pathways involved, with the aim of targeting them. Additionally, in future studies, one should seek to confirm these two genes as biomarkers of poorer prognosis and more aggressive or resistant tumors in patient specimens using, e.g., immunohistochemistry.

## 4. Materials and Methods

### 4.1. Data Preprocessing

In this study, we used multi-omics data from The Cancer Genome Atlas (TCGA) [11,12], considering 985 patients within the broader PanCancer initiative. We accessed six types of omics data for each BC patient via cBioPortal [27,28], including substitutions and small insertions/deletions, copy number alterations, methylation data, gene expression profiles, microRNA expression, and reverse-phase protein microarray (RPPA) data. The 146 metastatic BC cases [13] were also obtained from cBioPortal, where gene expression data were retrieved. A dataset comprising 1980 patients from the METABRIC database [24,25,26] was used to validate the previous results. Moreover, we also obtained and integrated clinical information such as OS for all the BC cases. Finally, we considered 150 samples from The Metastatic Breast Cancer Project (https://mbcproject.org/, accessed on 1 January 2022), also obtained from cBioPortal (https://www.cbioportal.org/study/summary?id=brca_mbcproject_2022, accessed on 1 January 2022).

### 4.2. Multi-Omics Integrative Clustering

To robustly identify a set of prognostic biomarkers, we first applied multi-omics clustering to group patients into biologically meaningful subtypes. This preliminary step not only reduces data dimensionality but also mitigates convergence issues—such as multicollinearity and an excessive number of predictors—that can compromise the performance of approaches based on regularized Cox regression.

To this end, we employed the Cancer Integration via MultIkernel LeaRning (CIMLR) [14] algorithm to integrate the six considered omics data types for subtype identification and patient stratification. CIMLR uses kernel-based machine learning to combine different data types, generating an integrated kernel matrix that captures patient similarity based on their molecular profiles. We calculated 385 Gaussian kernels for the seven data types and constructed a patient-to-patient similarity matrix. K-means clustering was then applied to this matrix, and the optimal number of clusters was determined using the elbow method.

### 4.3. Differential Analysis and Feature Selection

Continuous data were analyzed via analysis of variance (ANOVA) comparing distinct clusters. We applied the Benjamani–Hochberg correction to account for multiple testing and selected features with an adjusted *p*-value < 0.05.

### 4.4. Survival Analysis

We evaluated the prognostic significance of the identified clusters by associating them with overall survival (OS) over a 10-year period. Data points corresponding to patients who either died within one month of diagnosis or were older than 80 years were censored to avoid bias from uncertain observations. This exclusion criterion was applied to avoid bias from extreme cases that could distort survival estimates. Kaplan–Meier survival analysis with a log-rank test was used to assess the statistical significance of the associations, using a threshold of *p* < 0.05.

### 4.5. Regularized Cox Regression Analysis

We used the Coxnet algorithm [29,30] for regularized Cox regression analysis to identify significant predictors of patient outcomes. This method, a variant of the Cox proportional hazards model, applies a regularization term to shrink regression coefficients and select the most relevant variables. The elastic net method with the LASSO penalty was used to identify predictors with non-zero coefficients, selecting the model that minimized cross-validation error. Risk scores were calculated for each patient, based on a weighted sum of the selected covariates. Patients were stratified into different risk groups based on their risk scores relative to the dataset mean, providing insights into their prognosis.

### 4.6. RNA Extraction, Reverse Transcription and Real-Time Quantitative PCR

MCF-7pS and MCF-7pR cells were seeded in triplicate at a density of 3 × 10^6^ cells in T75 flasks and incubated to reach 70% confluence. Total RNA from the cells was extracted using RNeasy MiniKit (QIAGEN GmbH, Hilden, Germany) according to the manufacturer’s instructions. Total RNA (2 µg) was retrotranscribed with PrimeScript RT-Master Mix (Takara BIO Europe, SAS) in a 40 µL reaction volume. Of the resulting first-strand cDNA, 2 µL were amplified with Mastermix 2X (GeneSpin, Milan, Italy) in triplicate using a StepOnePlus™ Real-Time PCR System. Relative expression was normalized to the GAPDH using the 2^−ΔCt^ method. The TaqMan assays used are Hs00231133_m1 (LMO1), Hs01022301_m1 (PRAME), and Hs04400416_m1 (RSPO2) (ThermoFisher Scientific, Milano, Italy).

## Data Availability

This study utilized multi-omics data from The Cancer Genome Atlas (TCGA), accessed via the cBioPortal and publicly available from https://www.cbioportal.org/study/summary?id=brca_tcga_pan_can_atlas_2018. Metastatic breast cancer cases and validation data from the METABRIC database were also downloaded from the cBioPortal, respectively, from https://www.cbioportal.org/study/summary?id=metastatic_solid_tumors_mich_2017 and https://www.cbioportal.org/study/summary?id=brca_metabric (accessed on 1 December 2024).

## Supplementary Materials

The supporting information can be downloaded at: https://www.mdpi.com/article/10.3390/ijms26051943/s1.
