# Supplementary material for: Prognostic Biomarkers in Breast Cancer via Multi-Omics Clustering Analysis
Source: Int J Mol Sci. 2025 Feb 24;26(5):1943. doi: 10.3390/ijms26051943 (PMC11900291; doi:10.3390/ijms26051943)
Supplement: Supplementary file 1 [file ijms-26-01943-s001.zip › Capture of Supplementary Figures.pdf]

Capture of Supplementary Figures, Table and Data.

Supplementary Figure S1. Survival analysis using regularized Cox regression identifies potential biomarkers linked to patient outcomes.

Supplementary Figure S2. *LMO1* correlation with the TCGA's annotated PAM50 subtypes.

Supplementary Figure S3. *PRAME* correlation with the TCGA's annotated PAM50 subtypes.

Supplementary Figure S4. *RSPO2* correlation with the TCGA's annotated PAM50 subtypes.

Supplementary Figure S5. PAM50 annotations per Cluster.

Supplementary Figure S6. *LMO1, PRAME*, and *RSPO2* relative expression analysis. Validation of the expression data of *LMO1, PRAME,* and *RSPO2* using RT-qPCR, in MCF-7, MCF-7pR and in BT549.

Supplementary Table S1. Significant Biomarkers in Breast Cancer.

Supplementary Data S1. Identification of 32 significant genes with regularized Cox regression analysis.

Supplementary Data S2. Expression of *LMO1*, *PRAME* and *RSPO2* in metastatic patients.
