# Supplementary material for: Prognostic Biomarkers in Breast Cancer via Multi-Omics Clustering Analysis
Source: Int J Mol Sci. 2025 Feb 24;26(5):1943. doi: 10.3390/ijms26051943 (PMC11900291; doi:10.3390/ijms26051943)
Supplement: Supplementary file 1 [file ijms-26-01943-s001.zip › Supp Data S1.pdf]

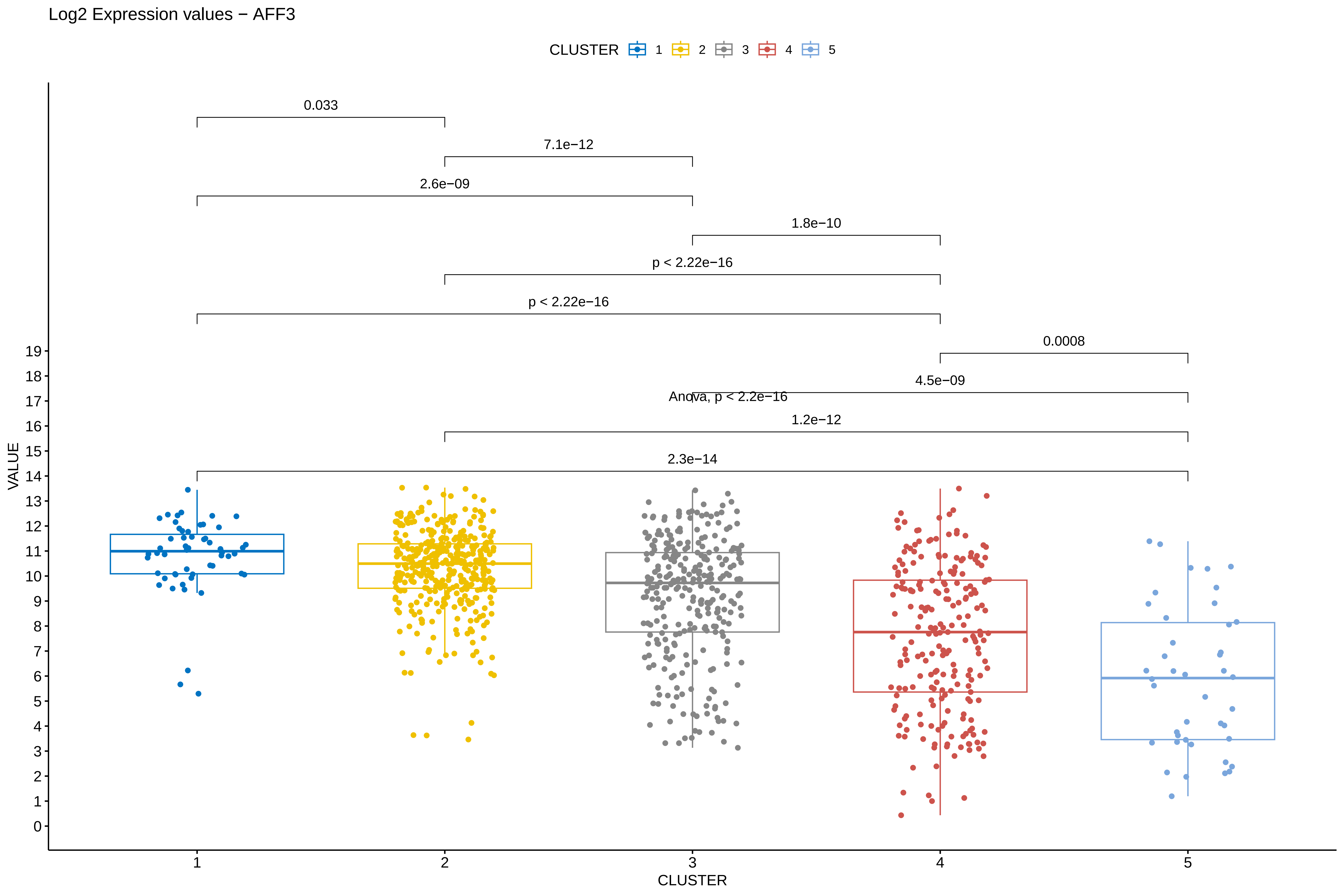

Log2 Expression values − AFF3
CLUSTER 1 2 3 4 5
0.033
7.1e−12
2.6e−09
1.8e−10
p < 2.22e−16
p < 2.22e−16
0.0008
4.5e−09
Anova, p < 2.2e−16
1.2e−12
2.3e−14
VALUE
0 1 2 3 4 5 6 7 8 9 10 11 12 13 14 15 16 17 18 19
1 2 3 4 5
CLUSTER

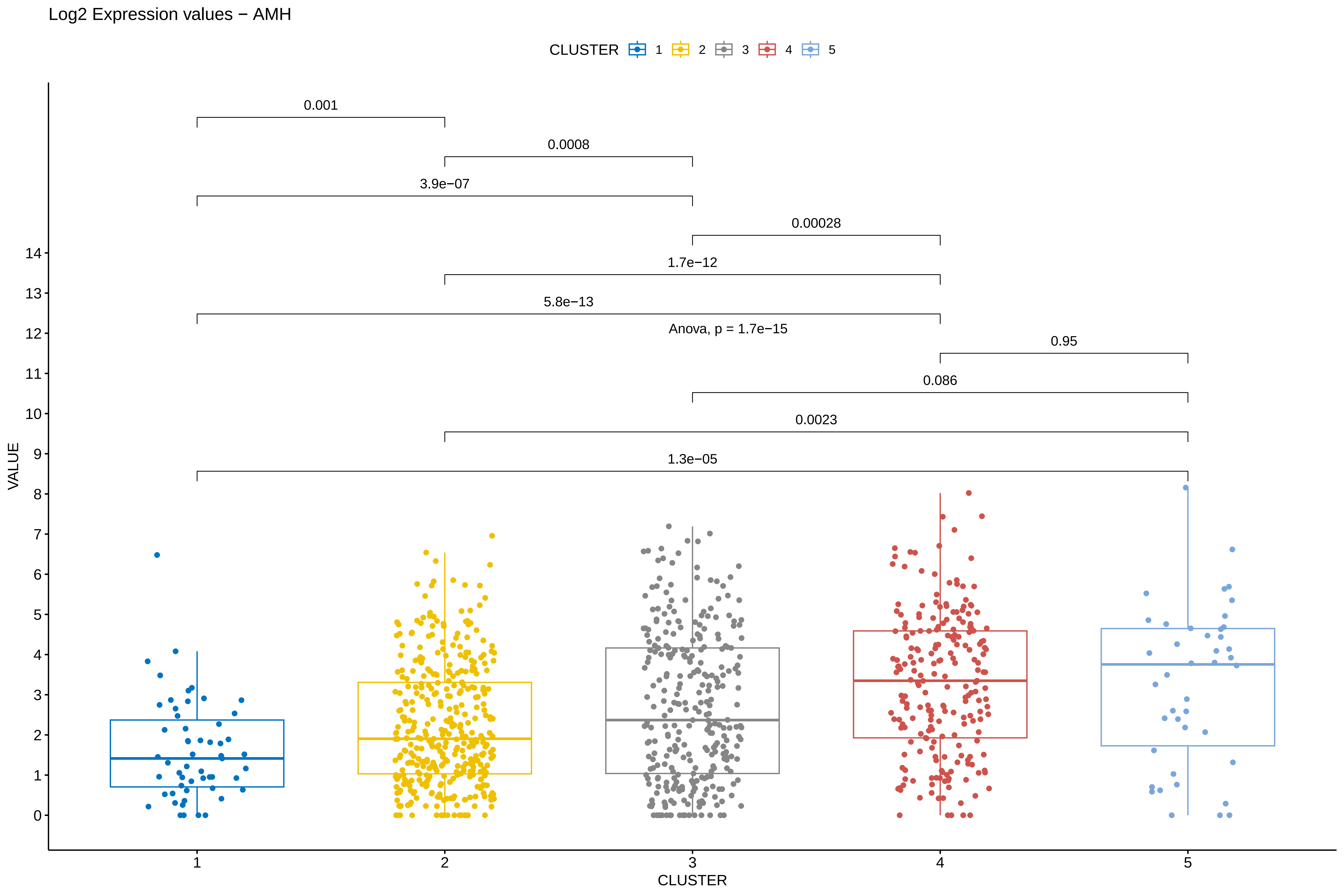
Log2 Expression values − AMH
CLUSTER 1 2 3 4 5
0.001
0.0008
3.9e−07
0.00028
1.7e−12
5.8e−13
Anova, p = 1.7e−15
0.95
0.086
0.0023
1.3e−05
VALUE
0
1
2
3
4
5
6
7
8
9
10
11
12
13
14
1
2
3
4
5
CLUSTER

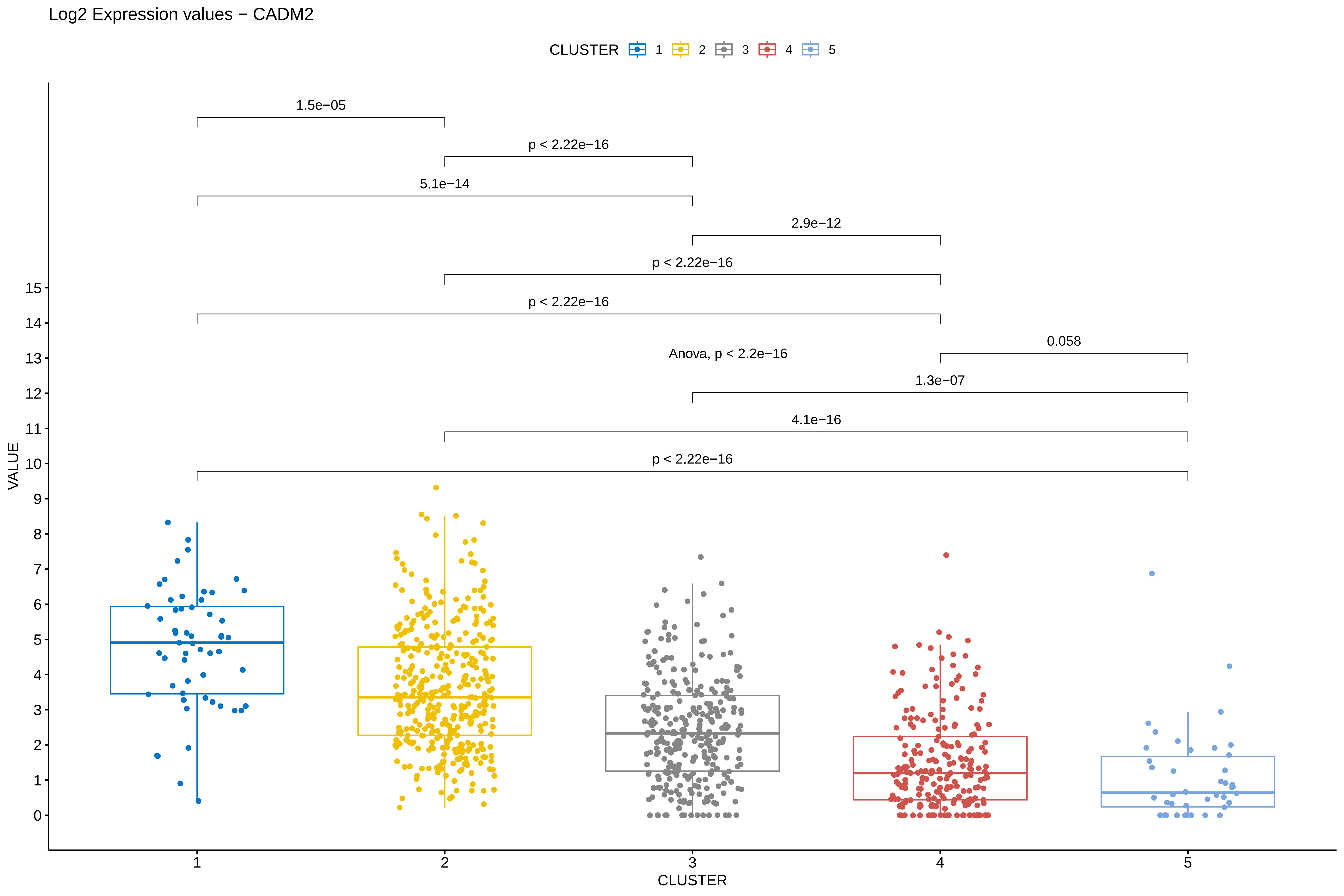
Log2 Expression values − CADM2
CLUSTER 1 2 3 4 5
1.5e−05
p < 2.22e−16
5.1e−14
2.9e−12
p < 2.22e−16
p < 2.22e−16
0.058
Anova, p < 2.2e−16
1.3e−07
4.1e−16
p < 2.22e−16
VALUE
0 1 2 3 4 5 6 7 8 9 10 11 12 13 14 15
1 2 3 4 5
CLUSTER

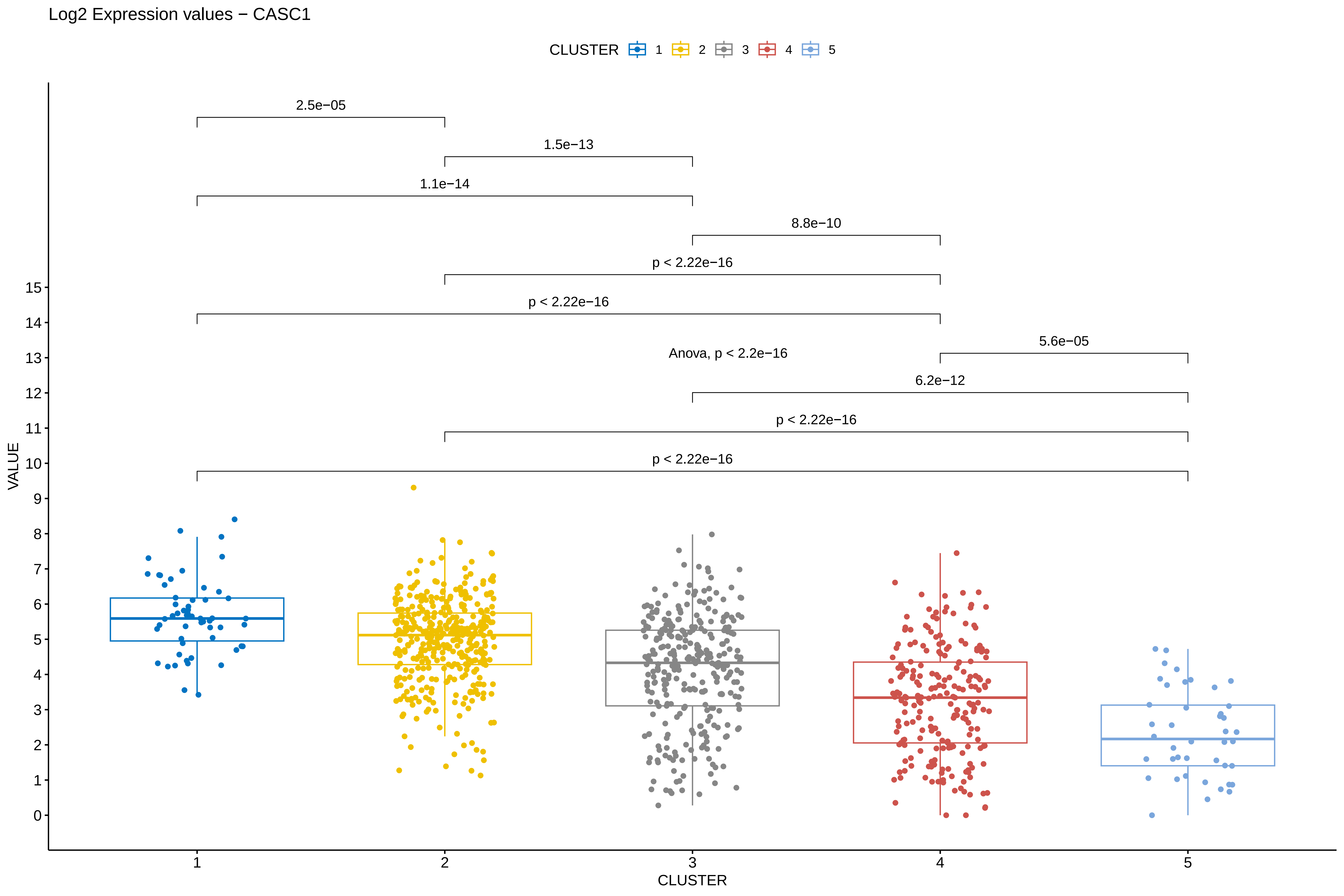

Log2 Expression values − CASC1
CLUSTER 1 2 3 4 5
2.5e−05
1.5e−13
1.1e−14
8.8e−10
p < 2.22e−16
p < 2.22e−16
5.6e−05
Anova, p < 2.2e−16
6.2e−12
p < 2.22e−16
p < 2.22e−16
VALUE
0 1 2 3 4 5 6 7 8 9 10 11 12 13 14 15
CLUSTER
1 2 3 4 5

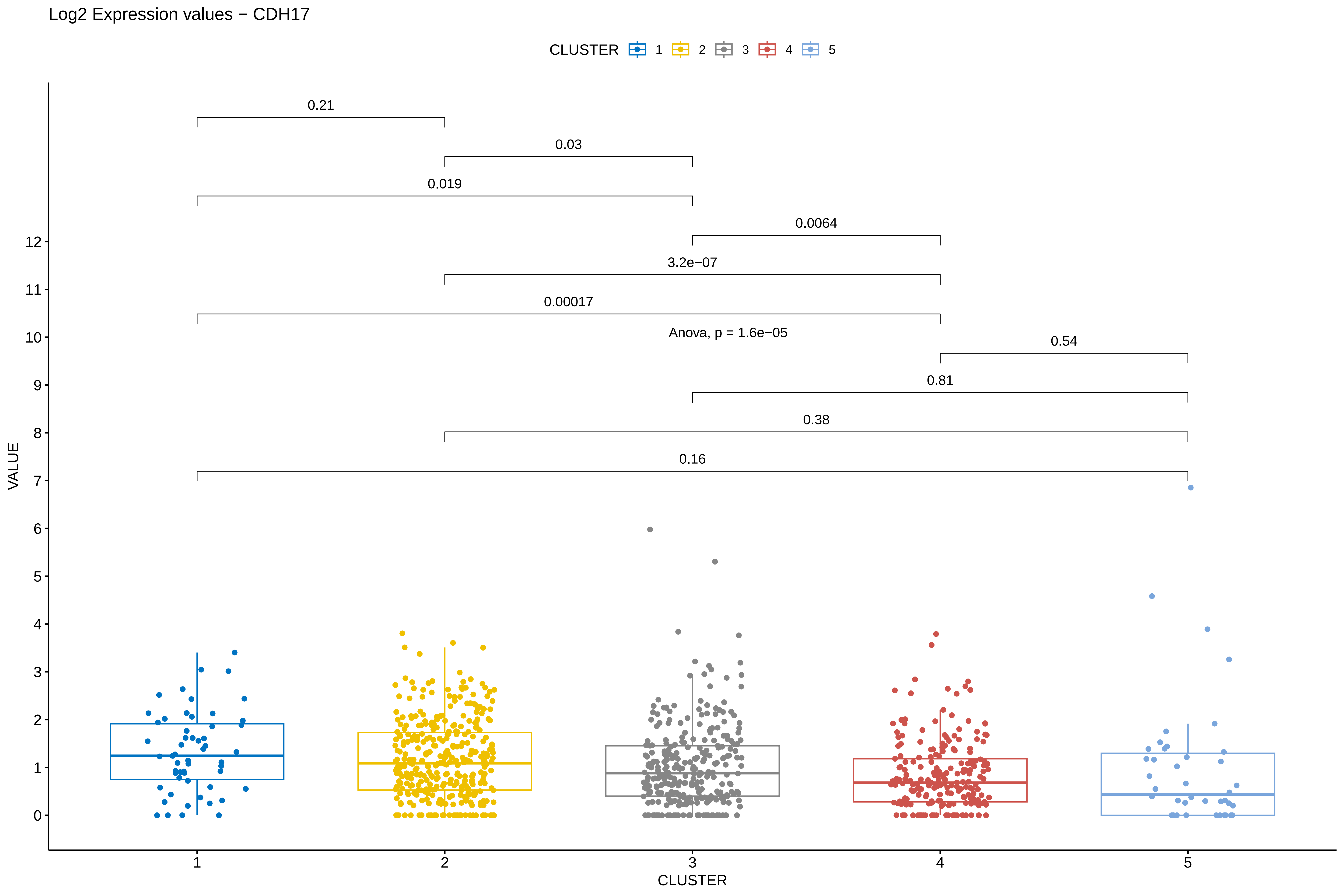
Log2 Expression values − CDH17
CLUSTER 1 2 3 4 5
0.21
0.03
0.019
0.0064
3.2e−07
0.00017
Anova, p = 1.6e−05
0.54
0.81
0.38
0.16
VALUE
0
1
2
3
4
5
6
7
8
9
10
11
12
1
2
3
4
5
CLUSTER

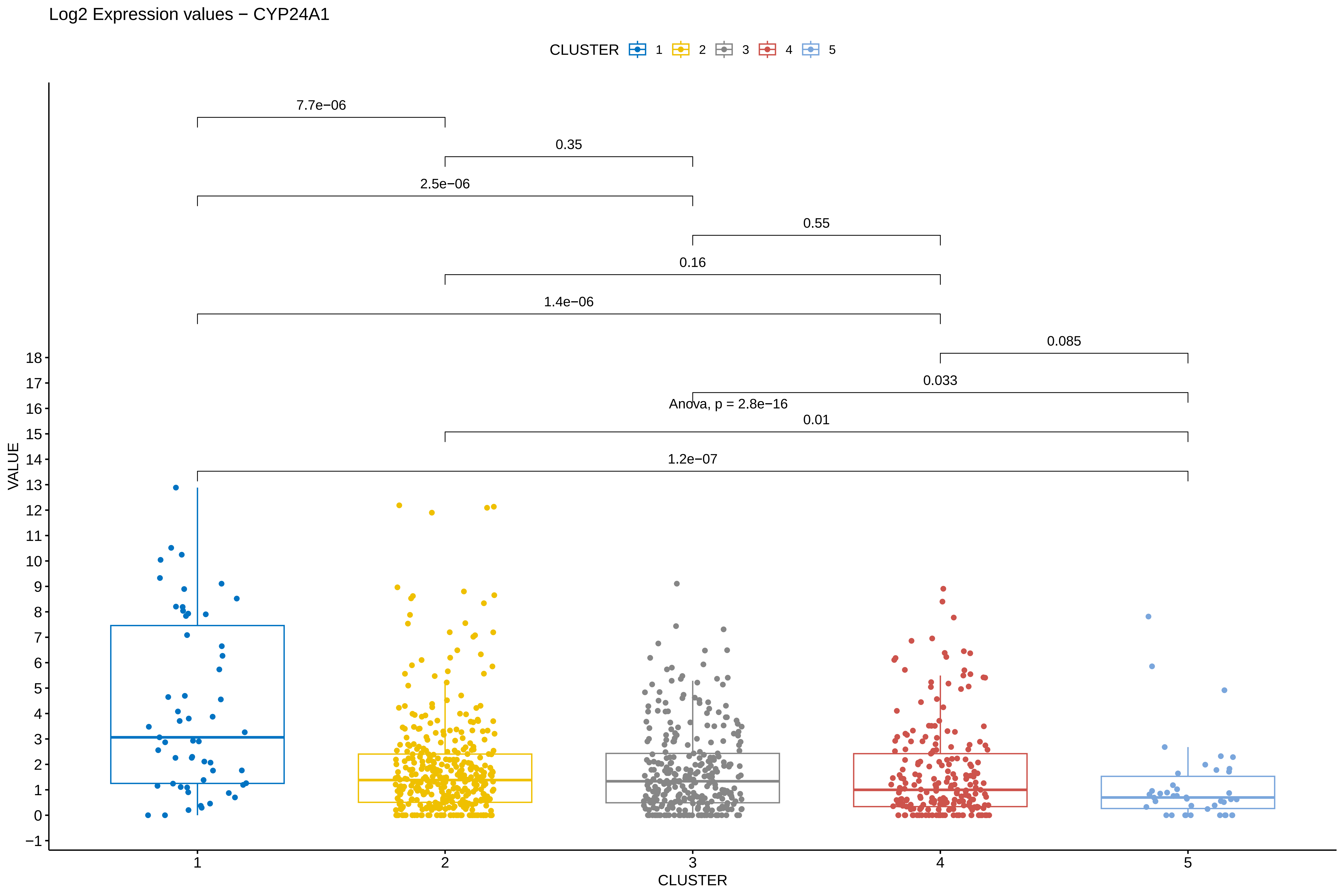

Log2 Expression values − CYP24A1
CLUSTER 1 2 3 4 5
7.7e−06
0.35
2.5e−06
0.55
0.16
1.4e−06
0.085
0.033
Anova, p = 2.8e−16
0.01
1.2e−07
VALUE
18
17
16
15
14
13
12
11
10
9
8
7
6
5
4
3
2
1
0
−1
1
2
3
4
5
CLUSTER

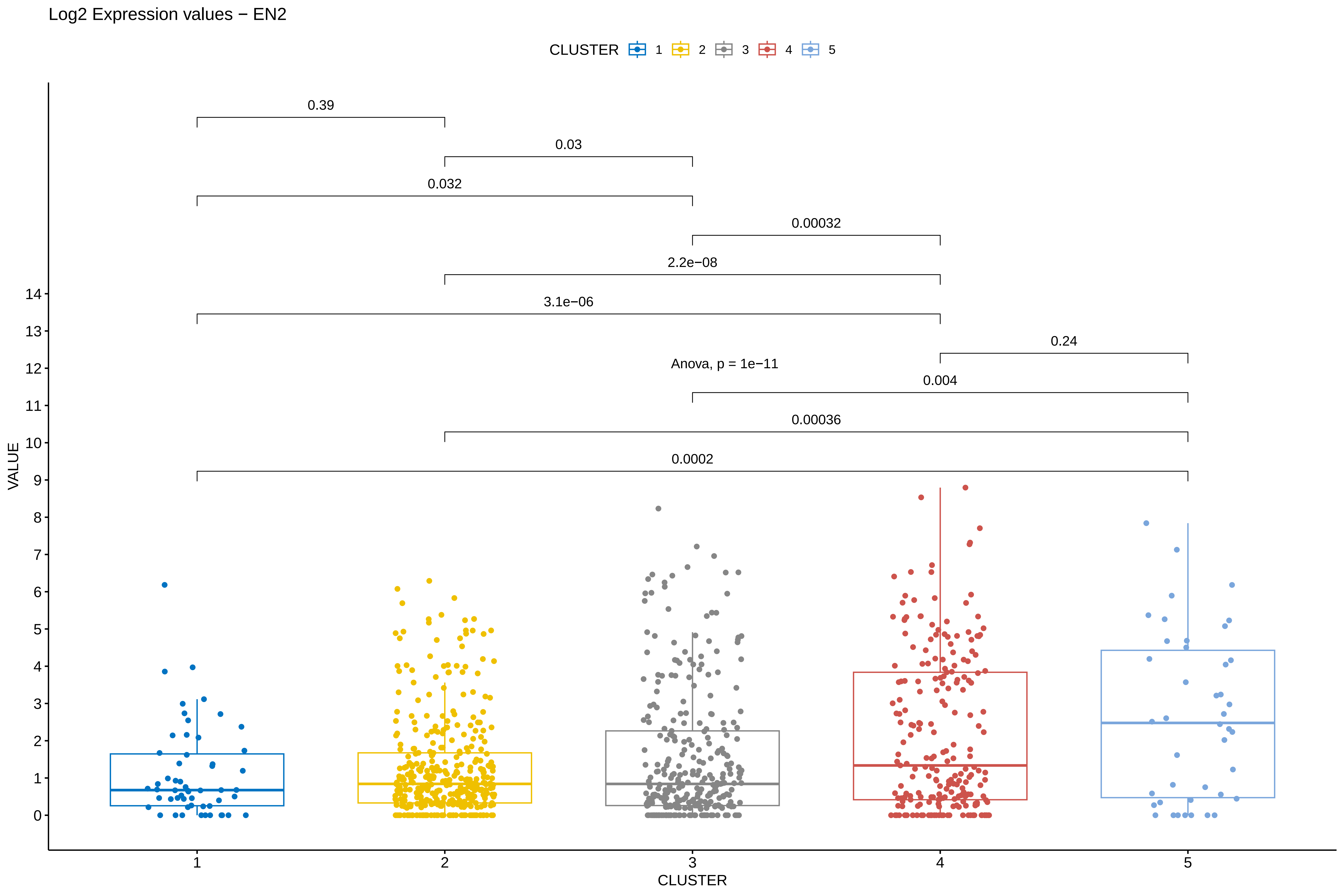
Log2 Expression values − EN2
CLUSTER 1 2 3 4 5
0.39
0.03
0.032
0.00032
2.2e−08
3.1e−06
0.24
Anova, p = 1e−11
0.004
0.00036
0.0002
VALUE
0
1
2
3
4
5
6
7
8
9
10
11
12
13
14
1
2
3
4
5
CLUSTER

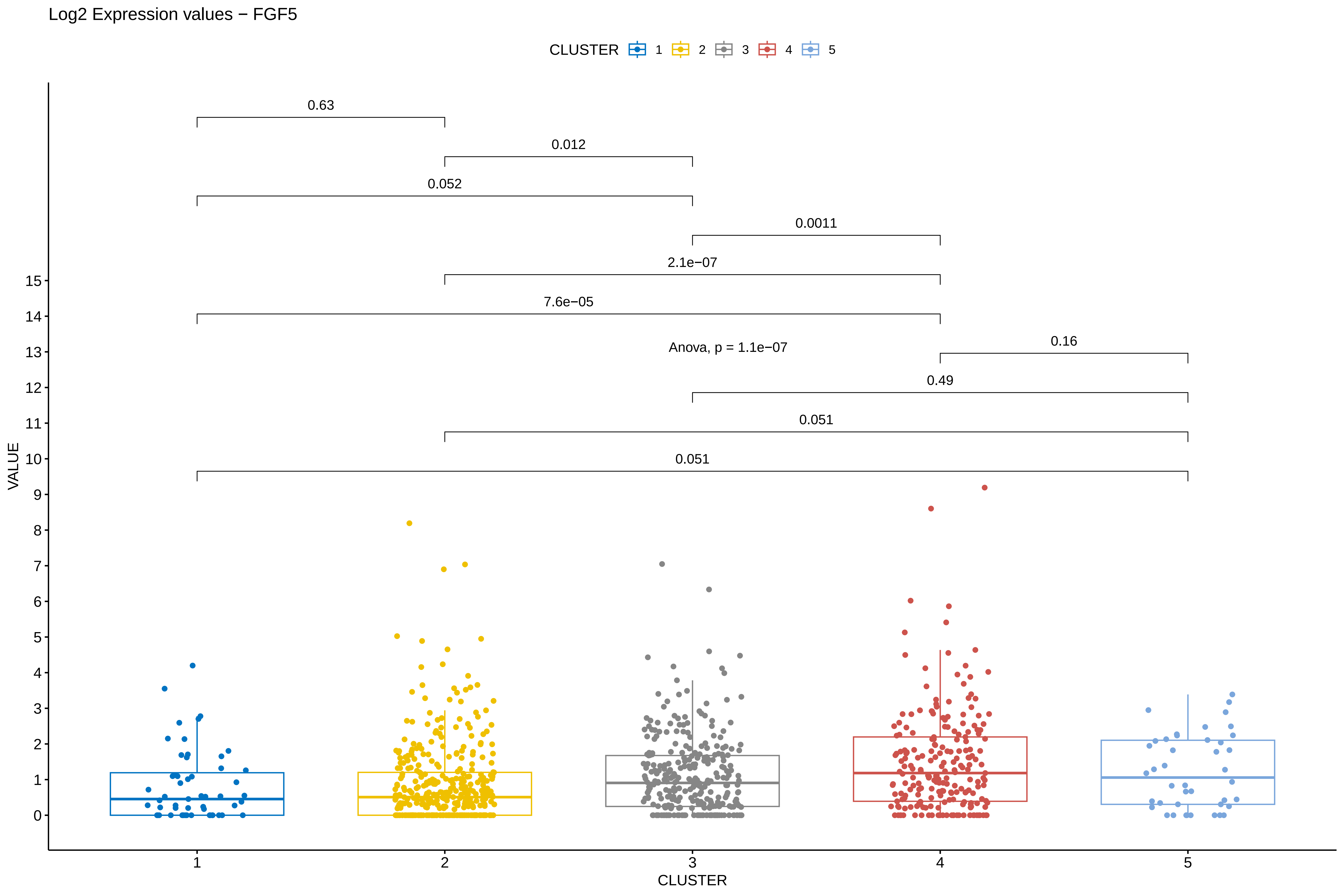

Log2 Expression values − FGF5
CLUSTER 1 2 3 4 5
0.63
0.012
0.052
0.0011
2.1e−07
7.6e−05
Anova, p = 1.1e−07
0.16
0.49
0.051
0.051
VALUE
0 1 2 3 4 5 6 7 8 9 10 11 12 13 14 15
1 2 3 4 5
CLUSTER

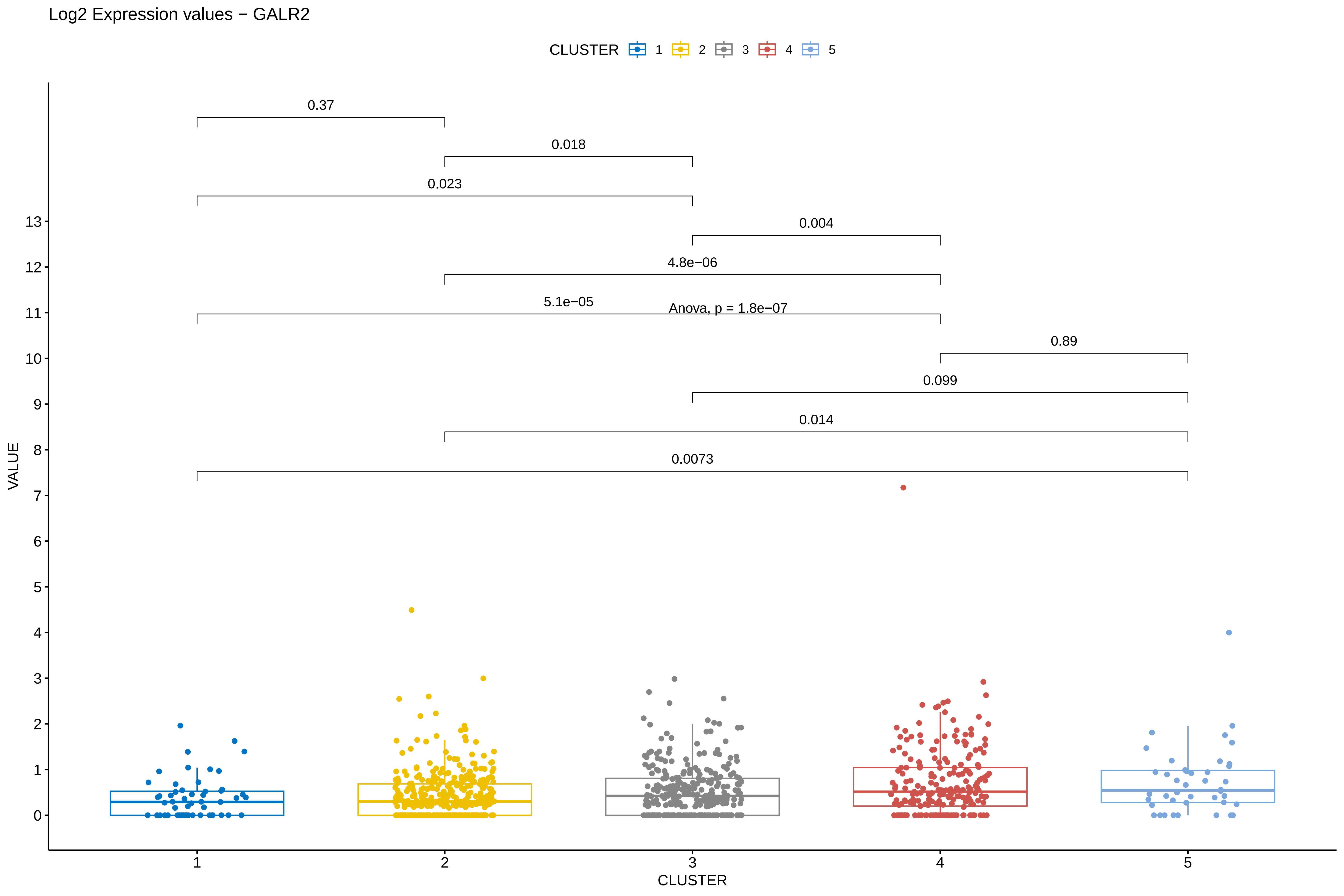

Log2 Expression values − GALR2
CLUSTER 1 2 3 4 5
0.37
0.018
0.023
0.004
4.8e−06
5.1e−05
Anova, p = 1.8e−07
0.89
0.099
0.014
0.0073
VALUE
0
1
2
3
4
5
6
7
8
9
10
11
12
13
1
2
3
4
5
CLUSTER

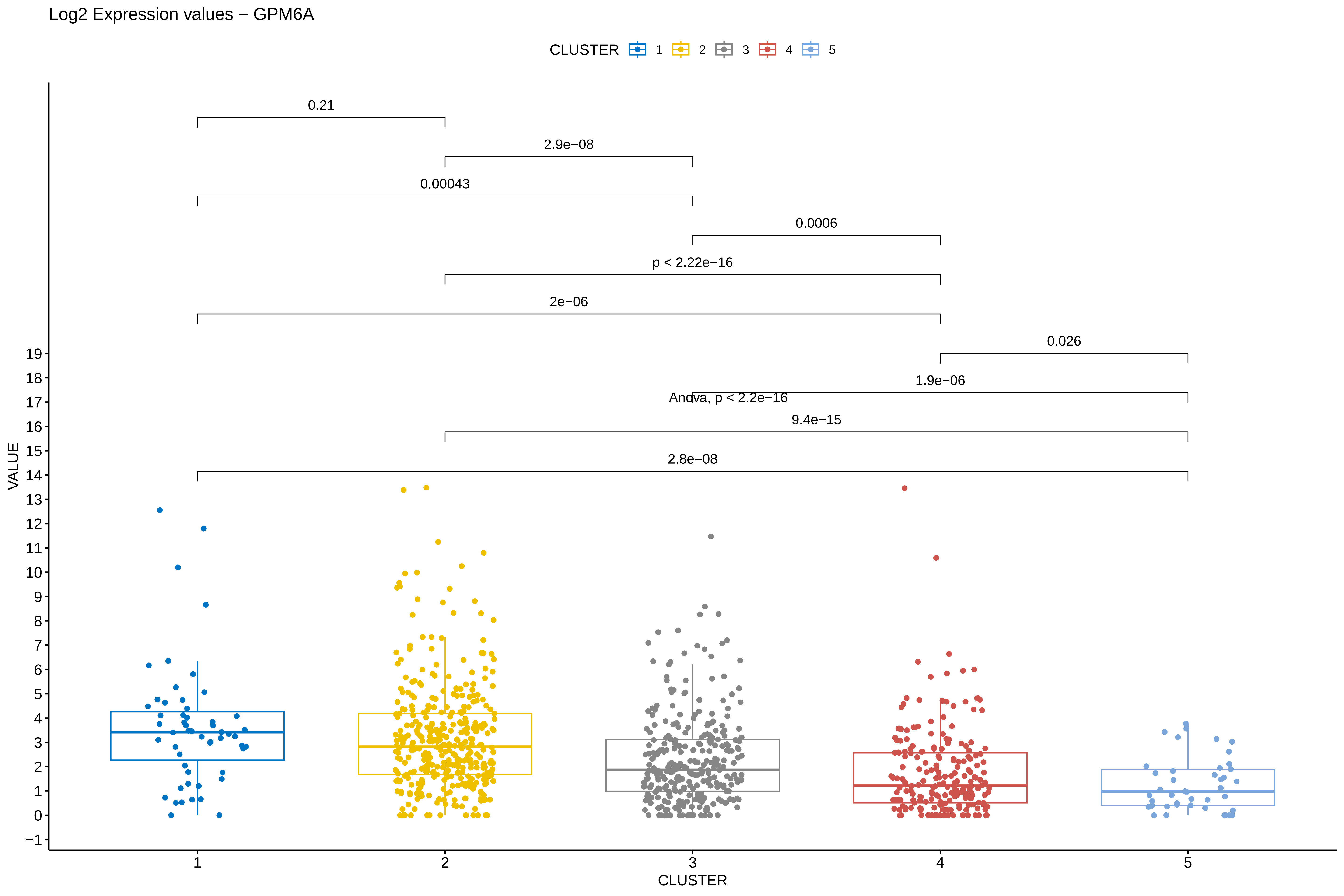

Log2 Expression values − GPM6A
CLUSTER 1 2 3 4 5
0.21
2.9e−08
0.00043
0.0006
p < 2.22e−16
2e−06
0.026
1.9e−06
Anova, p < 2.2e−16
9.4e−15
2.8e−08
VALUE
19
18
17
16
15
14
13
12
11
10
9
8
7
6
5
4
3
2
1
0
−1
1
2
3
4
5
CLUSTER

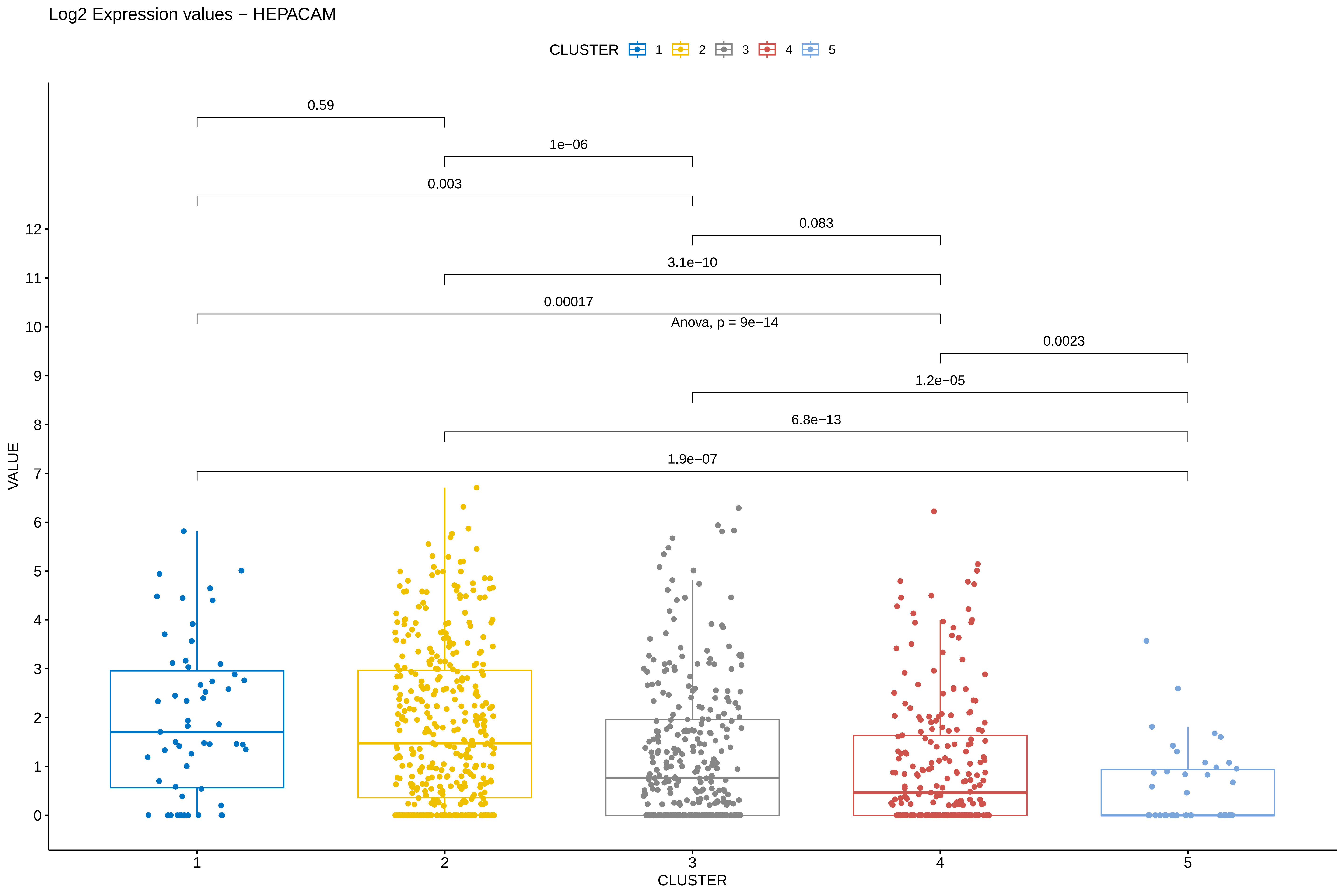

Log2 Expression values − HEPACAM
CLUSTER 1 2 3 4 5
0.59
1e−06
0.003
0.083
3.1e−10
0.00017
Anova, p = 9e−14
0.0023
1.2e−05
6.8e−13
1.9e−07
VALUE
0 1 2 3 4 5 6 7 8 9 10 11 12
1 2 3 4 5
CLUSTER

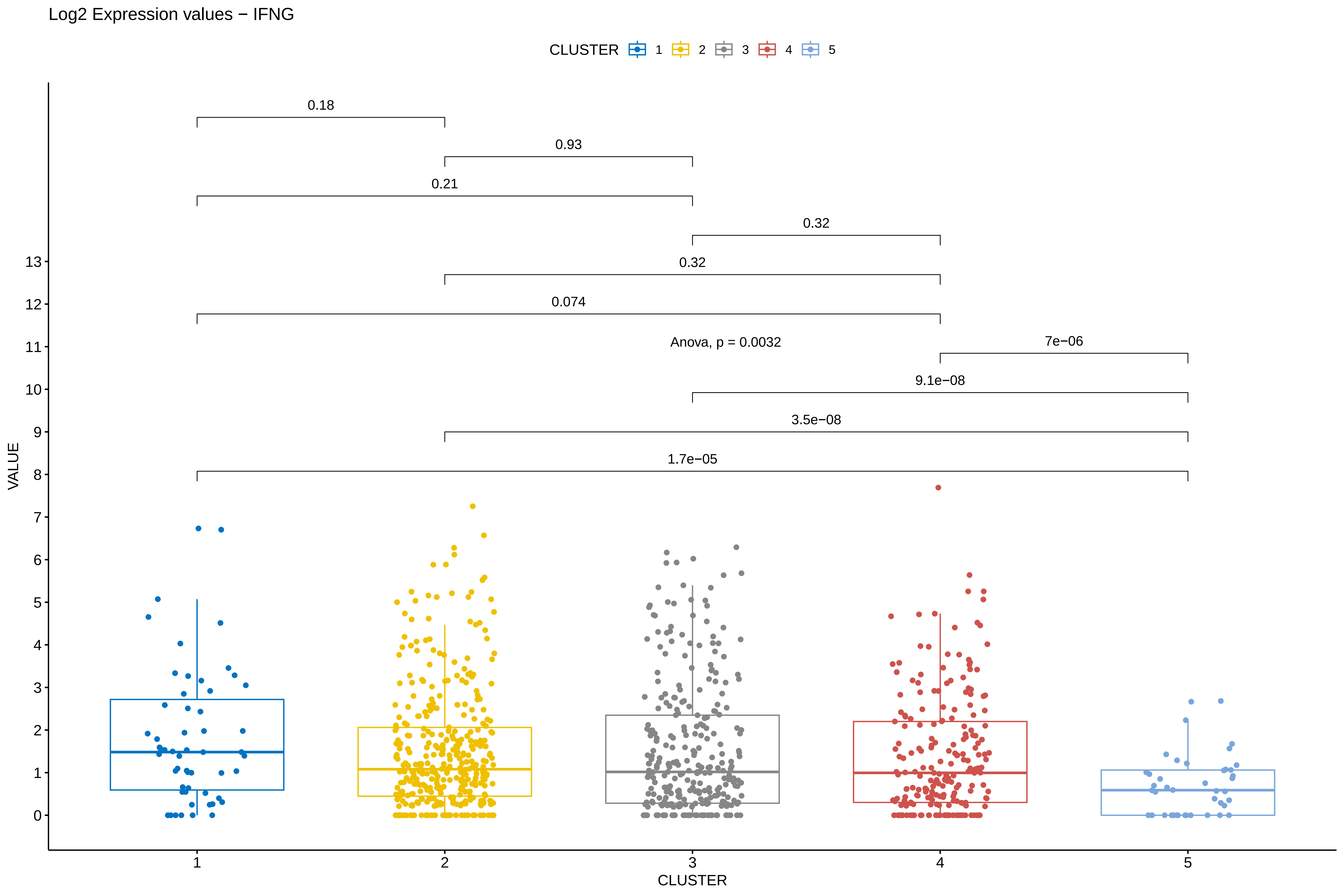
Log2 Expression values − IFNG
CLUSTER 1 2 3 4 5
0.18
0.93
0.21
0.32
0.32
0.074
Anova, p = 0.0032
7e−06
9.1e−08
3.5e−08
1.7e−05
VALUE
0
1
2
3
4
5
6
7
8
9
10
11
12
13
1
2
3
4
5
CLUSTER

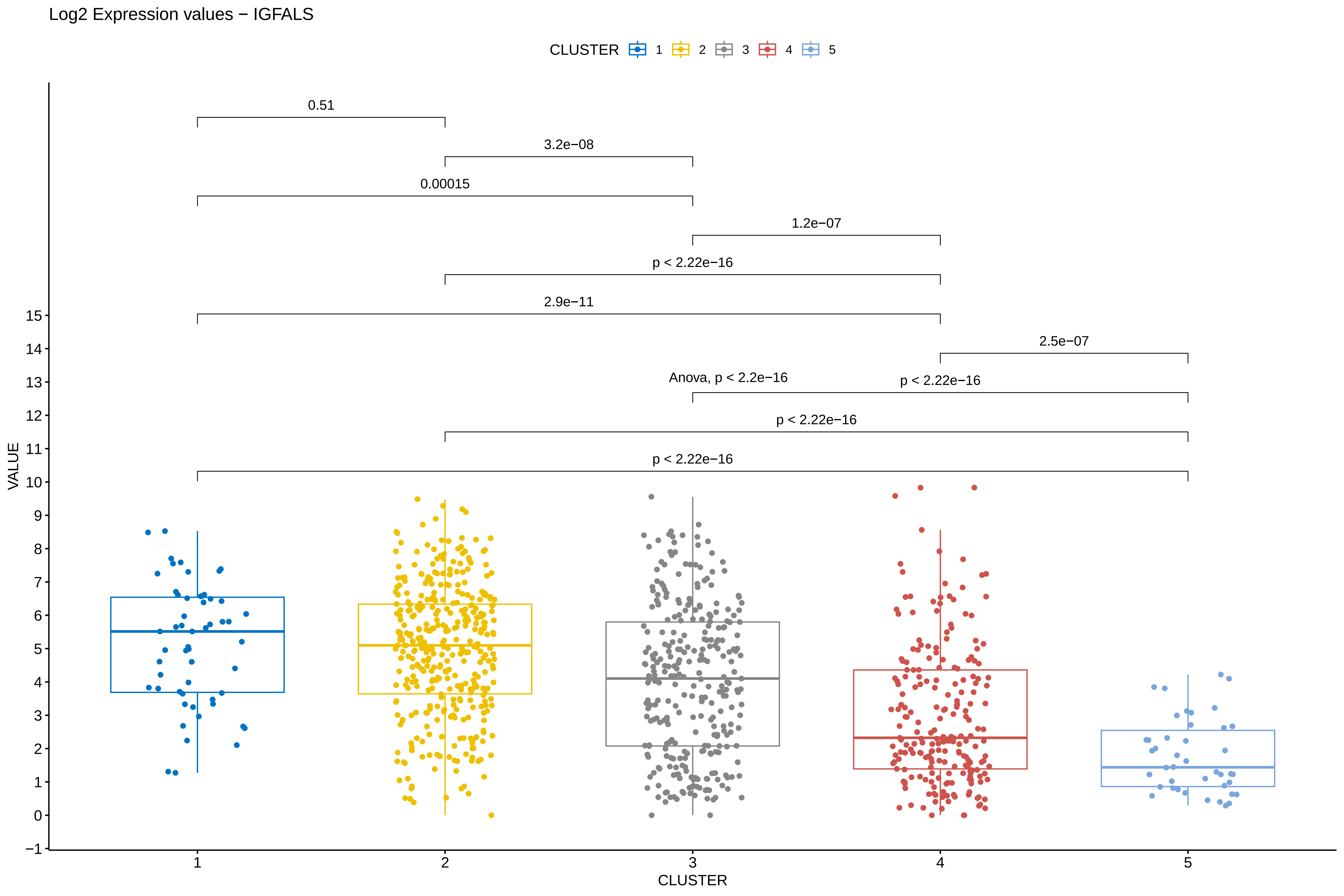
Log2 Expression values − IGFALS
CLUSTER 1 2 3 4 5
0.51
3.2e−08
0.00015
1.2e−07
p < 2.22e−16
2.9e−11
2.5e−07
Anova, p < 2.2e−16
p < 2.22e−16
p < 2.22e−16
p < 2.22e−16
VALUE
CLUSTER
−1 0 1 2 3 4 5 6 7 8 9 10 11 12 13 14 15
1 2 3 4 5

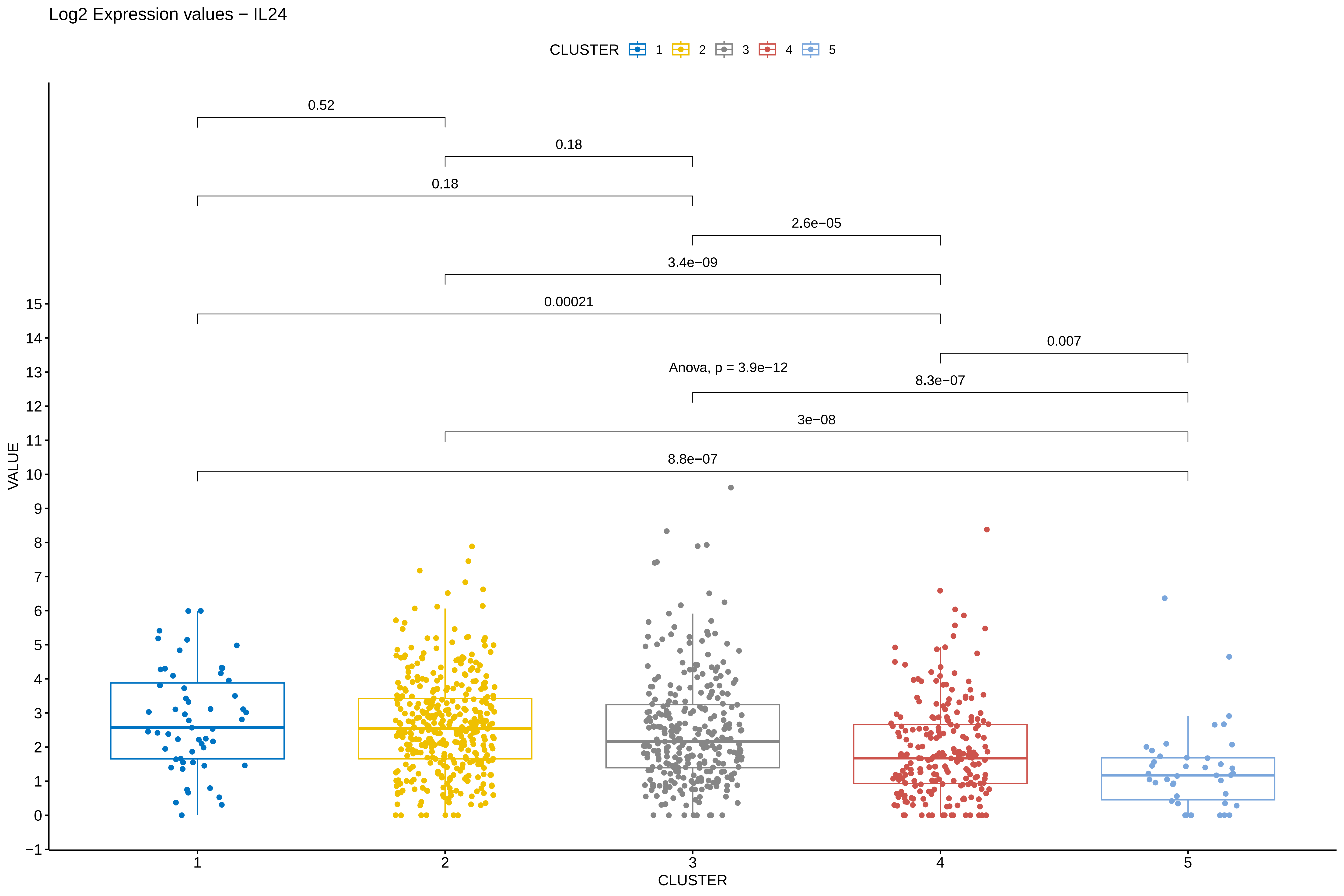

Log2 Expression values − IL24
CLUSTER 1 2 3 4 5
0.52
0.18
0.18
2.6e−05
3.4e−09
0.00021
0.007
Anova, p = 3.9e−12
8.3e−07
3e−08
8.8e−07
VALUE
15
14
13
12
11
10
9
8
7
6
5
4
3
2
1
0
−1
1
2
3
4
5
CLUSTER

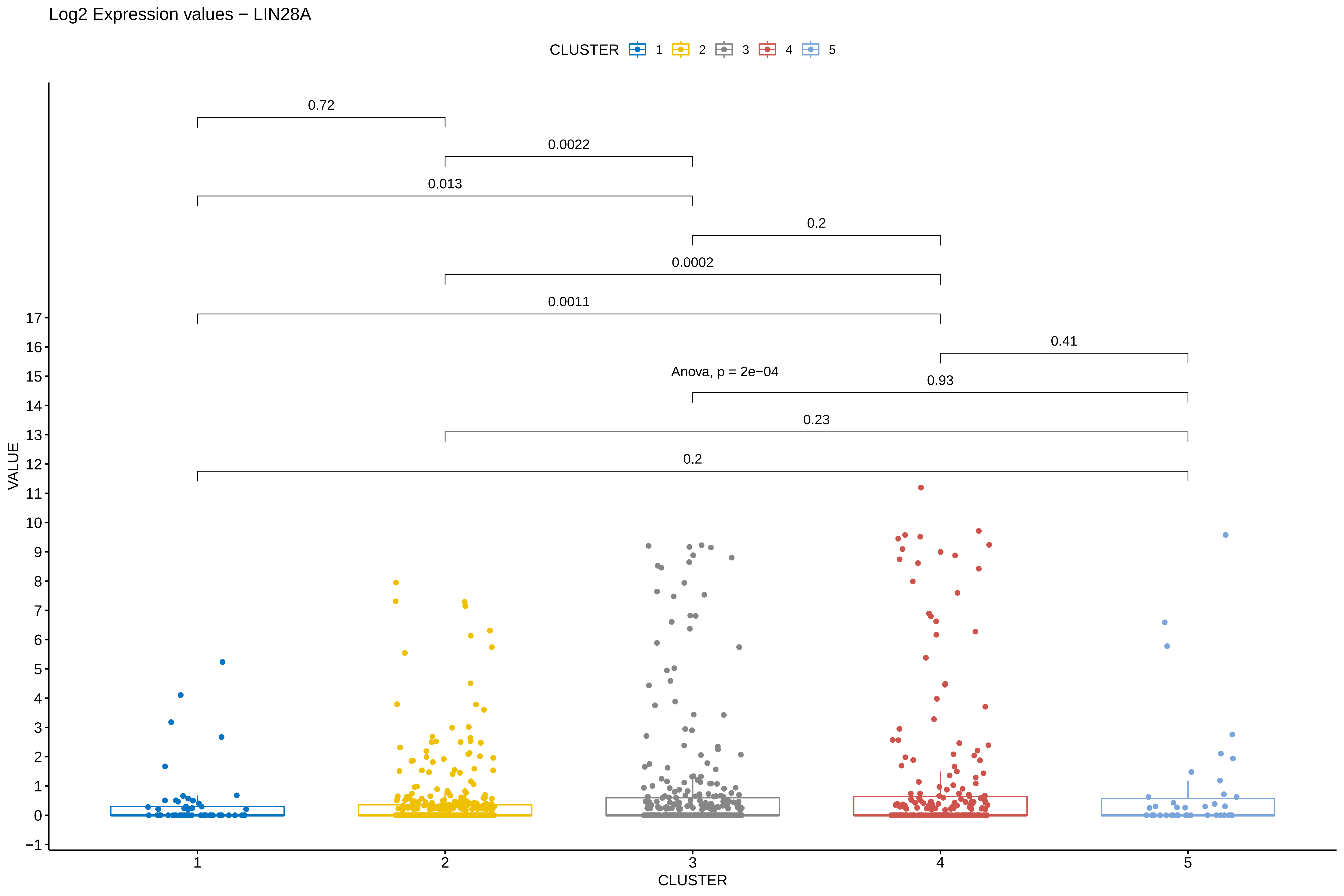

Log2 Expression values − LIN28A
CLUSTER 1 2 3 4 5
0.72
0.0022
0.013
0.2
0.0002
0.0011
0.41
Anova, p = 2e−04
0.93
0.23
0.2
VALUE
17
16
15
14
13
12
11
10
9
8
7
6
5
4
3
2
1
0
−1
1
2
3
4
5
CLUSTER

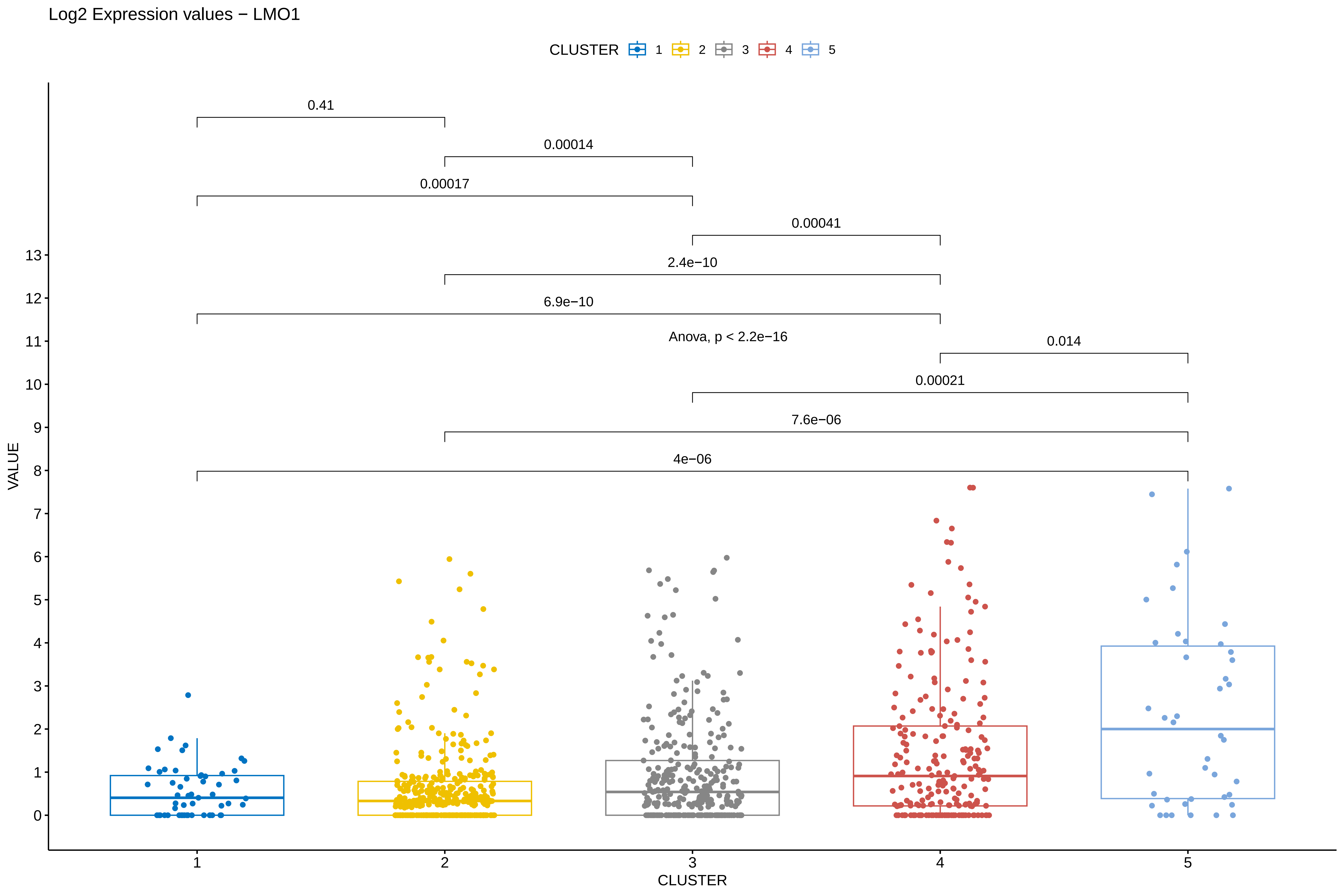

Log2 Expression values − LMO1
CLUSTER 1 2 3 4 5
0.41
0.00014
0.00017
0.00041
2.4e−10
6.9e−10
Anova, p < 2.2e−16
0.014
0.00021
7.6e−06
4e−06
VALUE
0
1
2
3
4
5
6
7
8
9
10
11
12
13
CLUSTER
1
2
3
4
5

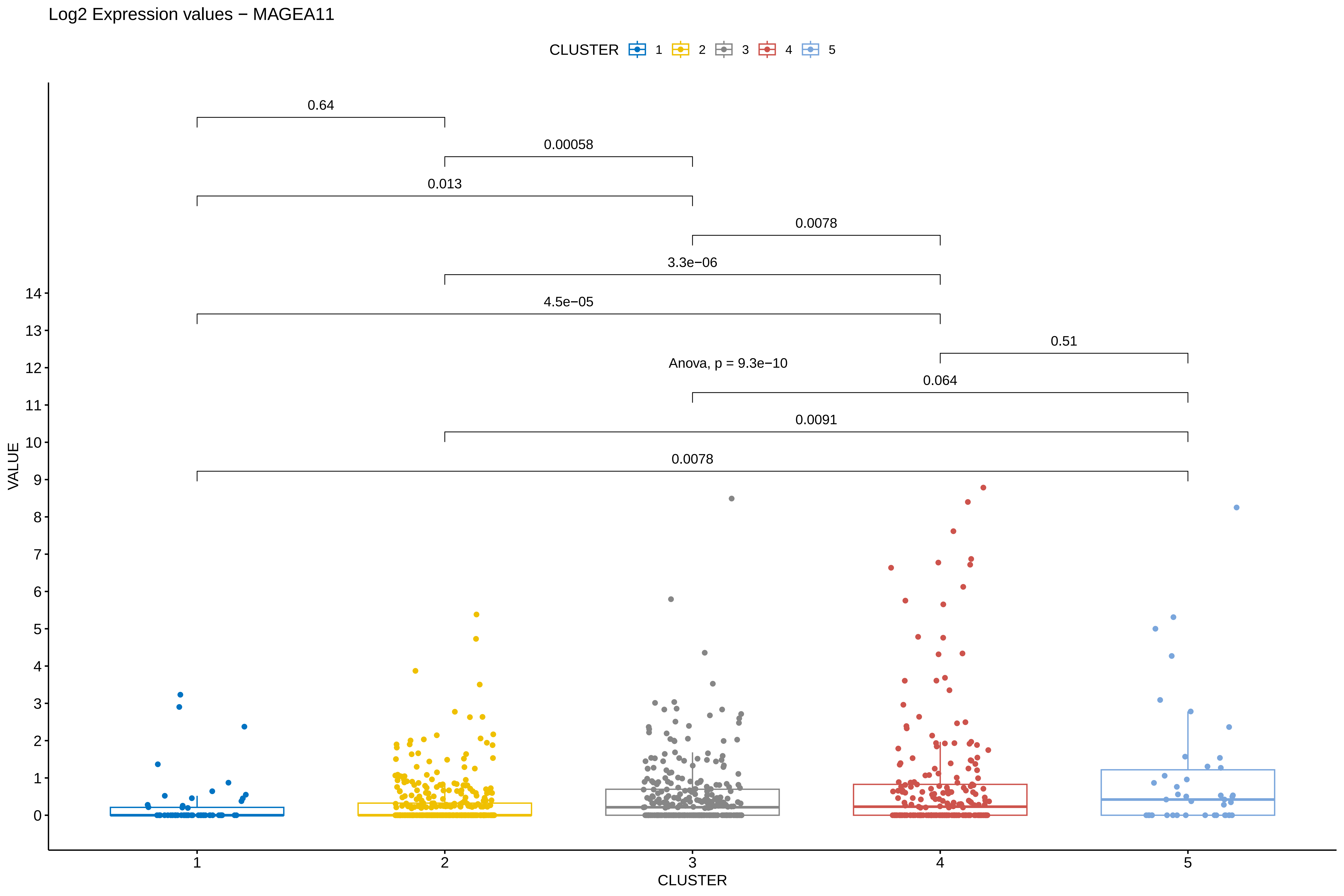
Log2 Expression values − MAGEA11
CLUSTER 1 2 3 4 5
0.64
0.00058
0.013
0.0078
3.3e−06
4.5e−05
0.51
Anova, p = 9.3e−10
0.064
0.0091
0.0078
VALUE
0
1
2
3
4
5
6
7
8
9
10
11
12
13
14
CLUSTER
1
2
3
4
5

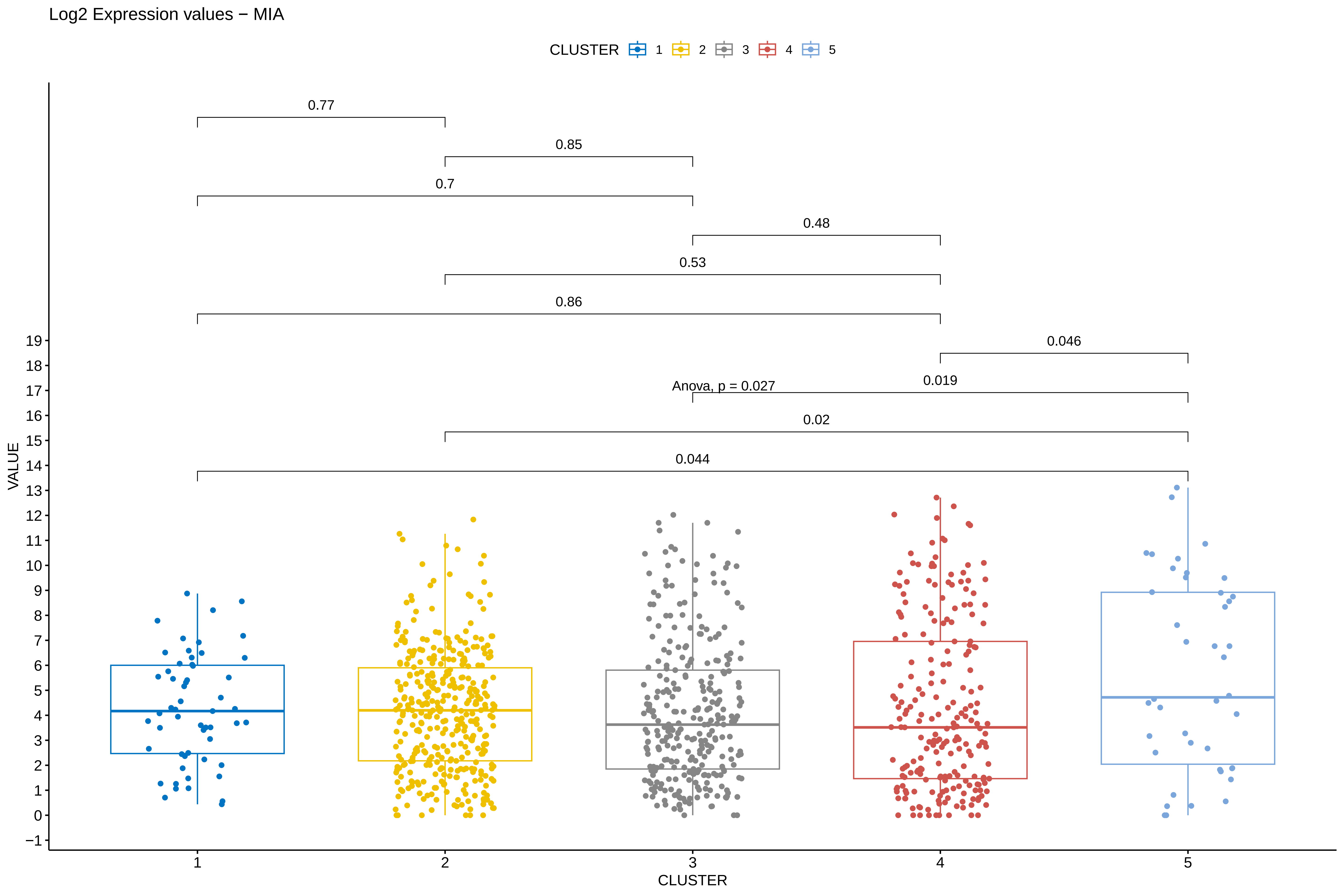

Log2 Expression values − MIA
CLUSTER 1 2 3 4 5
0.77
0.85
0.7
0.48
0.53
0.86
0.046
Anova, p = 0.027
0.019
0.02
0.044
VALUE
CLUSTER

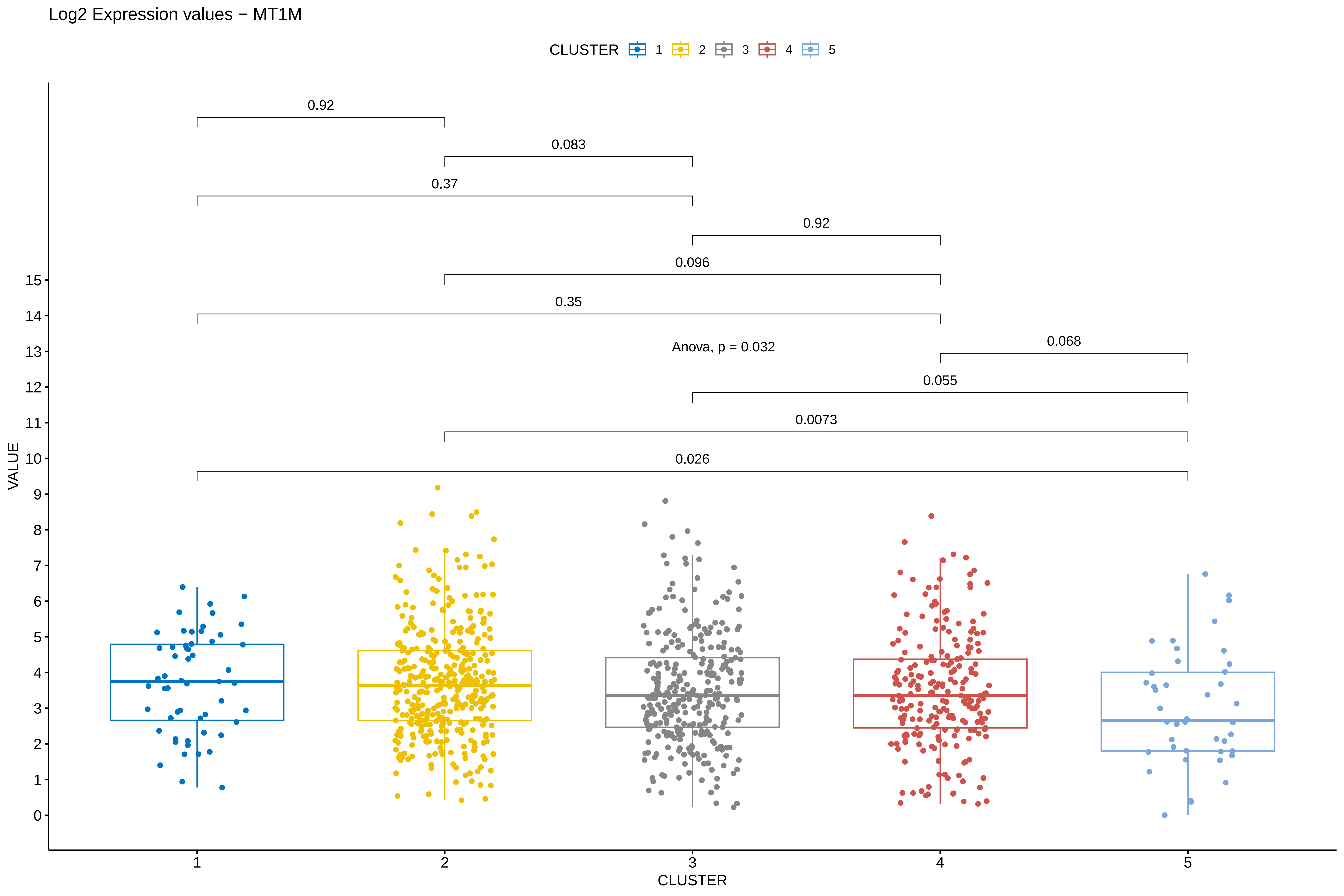

Log2 Expression values − MT1M
CLUSTER 1 2 3 4 5
0.92
0.083
0.37
0.92
0.096
0.35
Anova, p = 0.032
0.068
0.055
0.0073
0.026
VALUE
0
1
2
3
4
5
6
7
8
9
10
11
12
13
14
15
CLUSTER
1
2
3
4
5

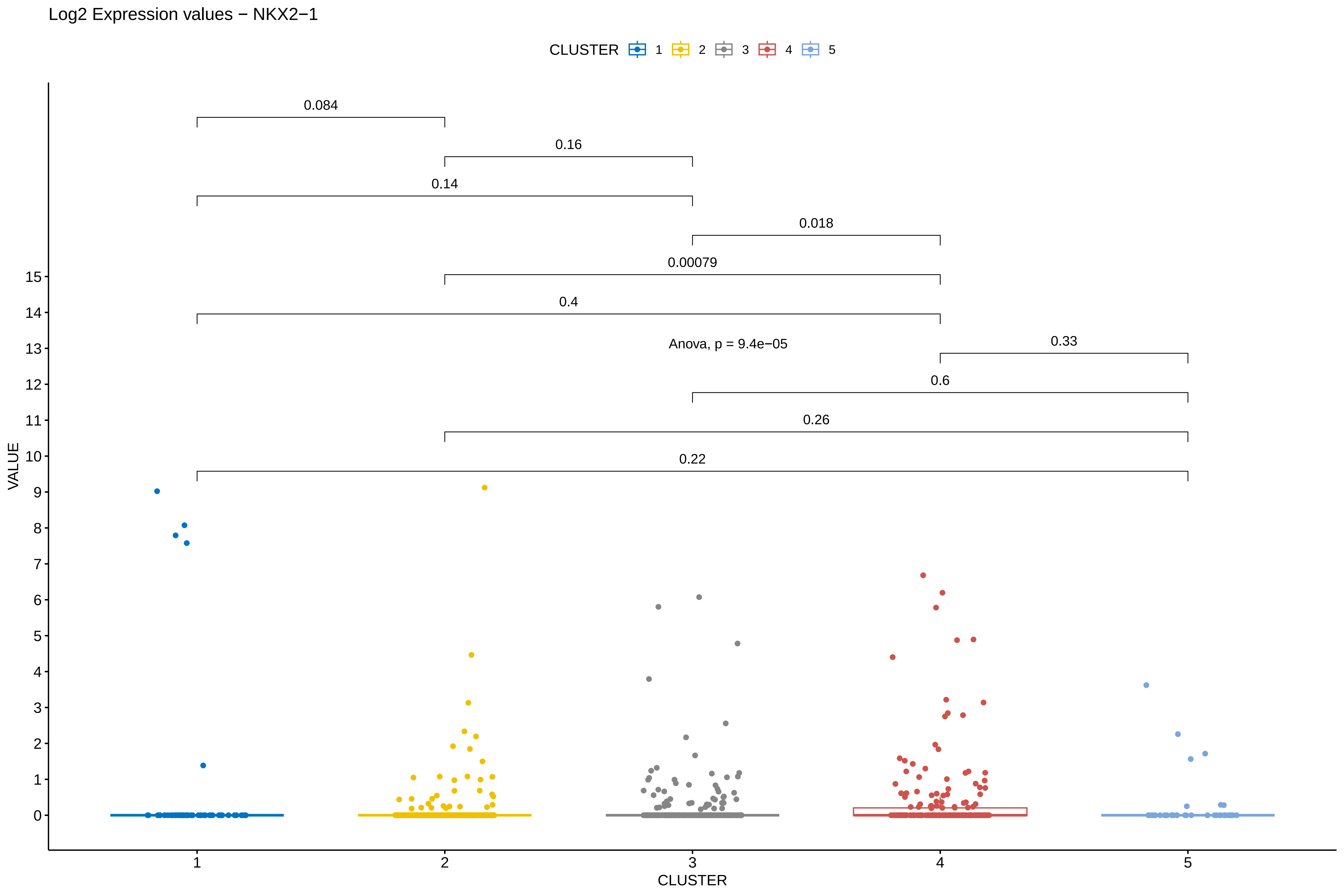

Log2 Expression values − NKX2−1
CLUSTER 1 2 3 4 5
0.084
0.16
0.14
0.018
0.00079
0.4
Anova, p = 9.4e−05
0.33
0.6
0.26
0.22
VALUE
15
14
13
12
11
10
9
8
7
6
5
4
3
2
1
0
1
2
3
4
5
CLUSTER

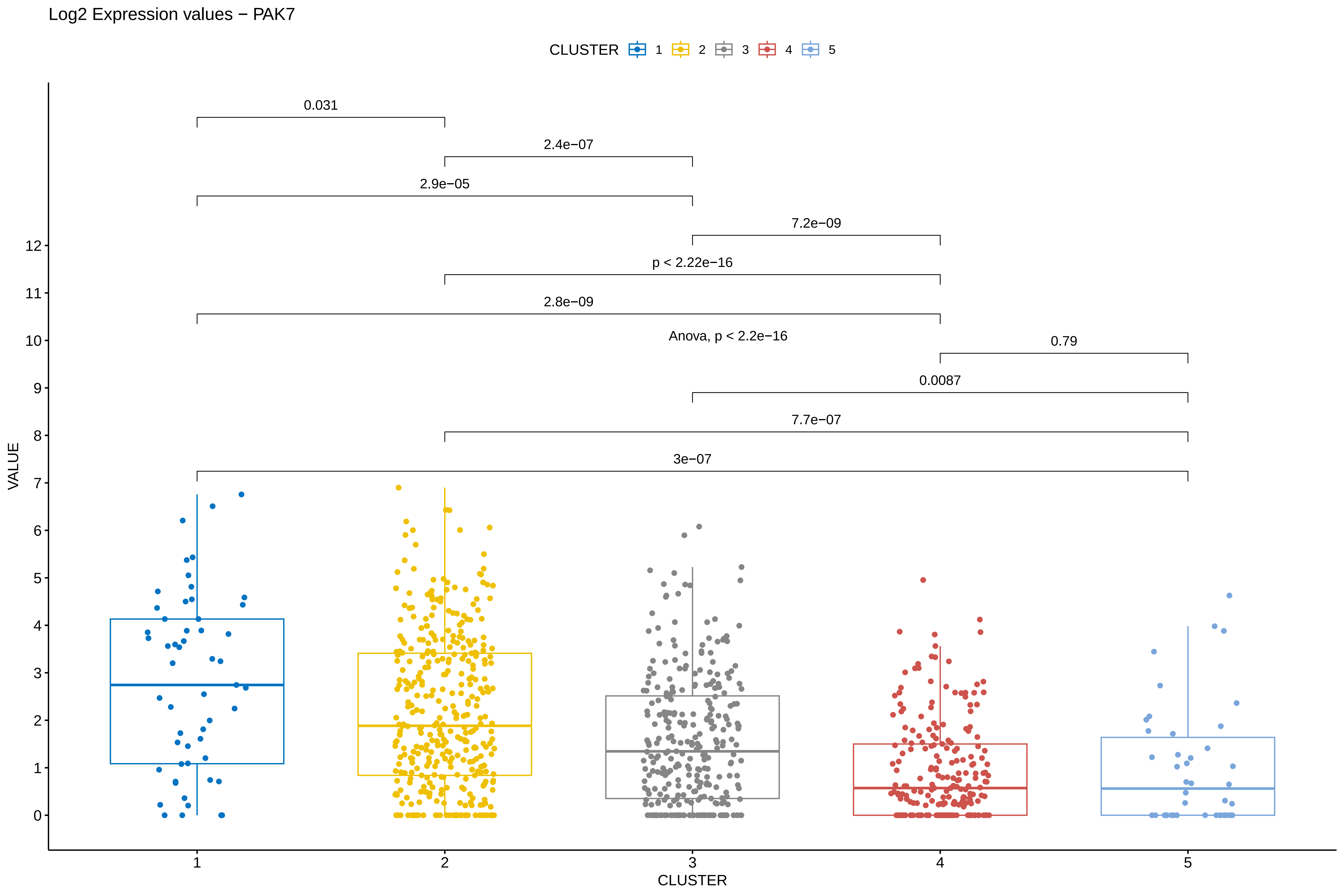
Log2 Expression values − PAK7
CLUSTER 1 2 3 4 5
0.031
2.4e−07
2.9e−05
7.2e−09
p < 2.22e−16
2.8e−09
Anova, p < 2.2e−16
0.79
0.0087
7.7e−07
3e−07
VALUE
0
1
2
3
4
5
6
7
8
9
10
11
12
CLUSTER
1
2
3
4
5

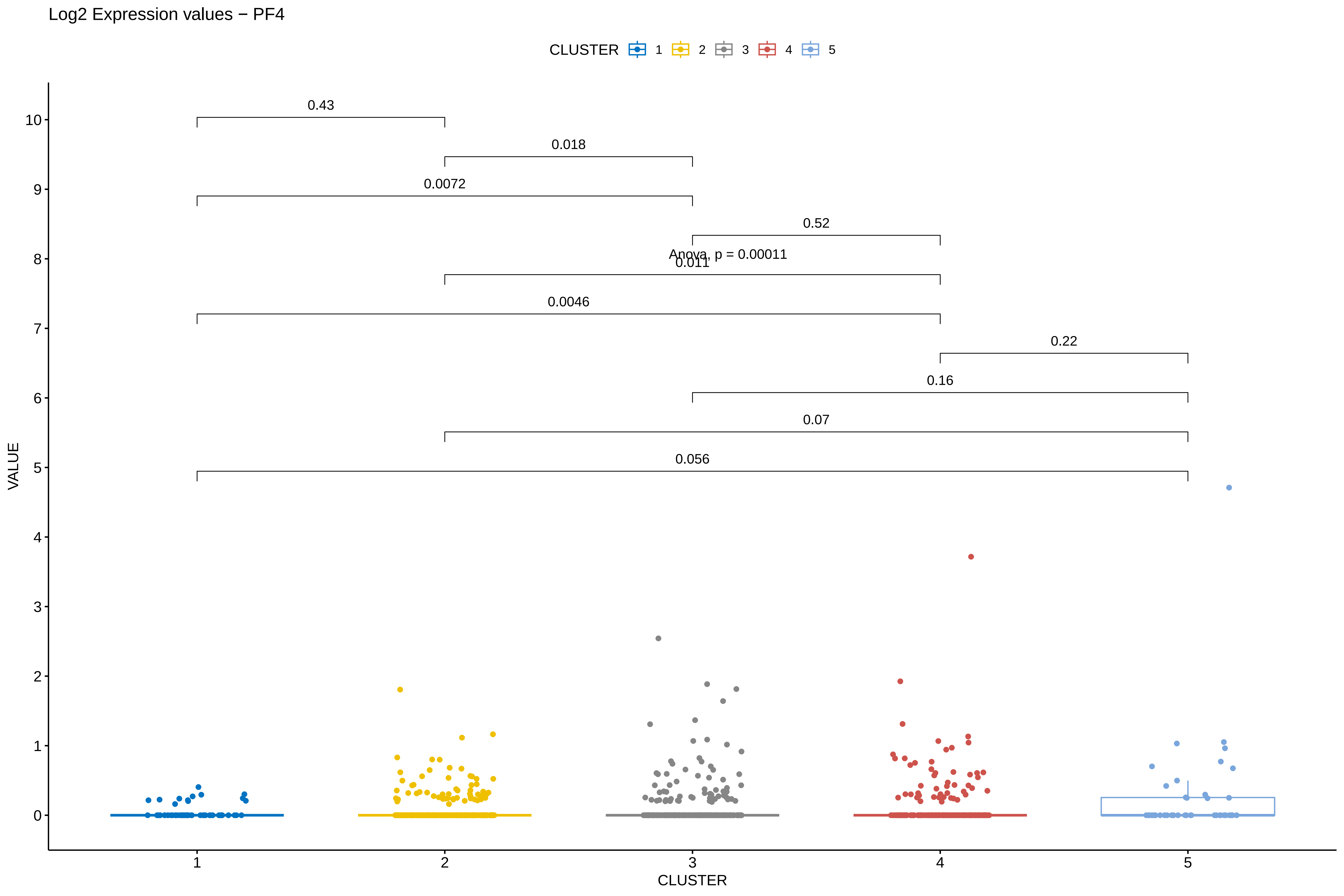

Log2 Expression values − PF4
CLUSTER 1 2 3 4 5
0.43
0.018
0.0072
0.52
Anova, p = 0.00011
0.011
0.0046
0.22
0.16
0.07
0.056
VALUE
0
1
2
3
4
5
6
7
8
9
10
1
2
3
4
5
CLUSTER

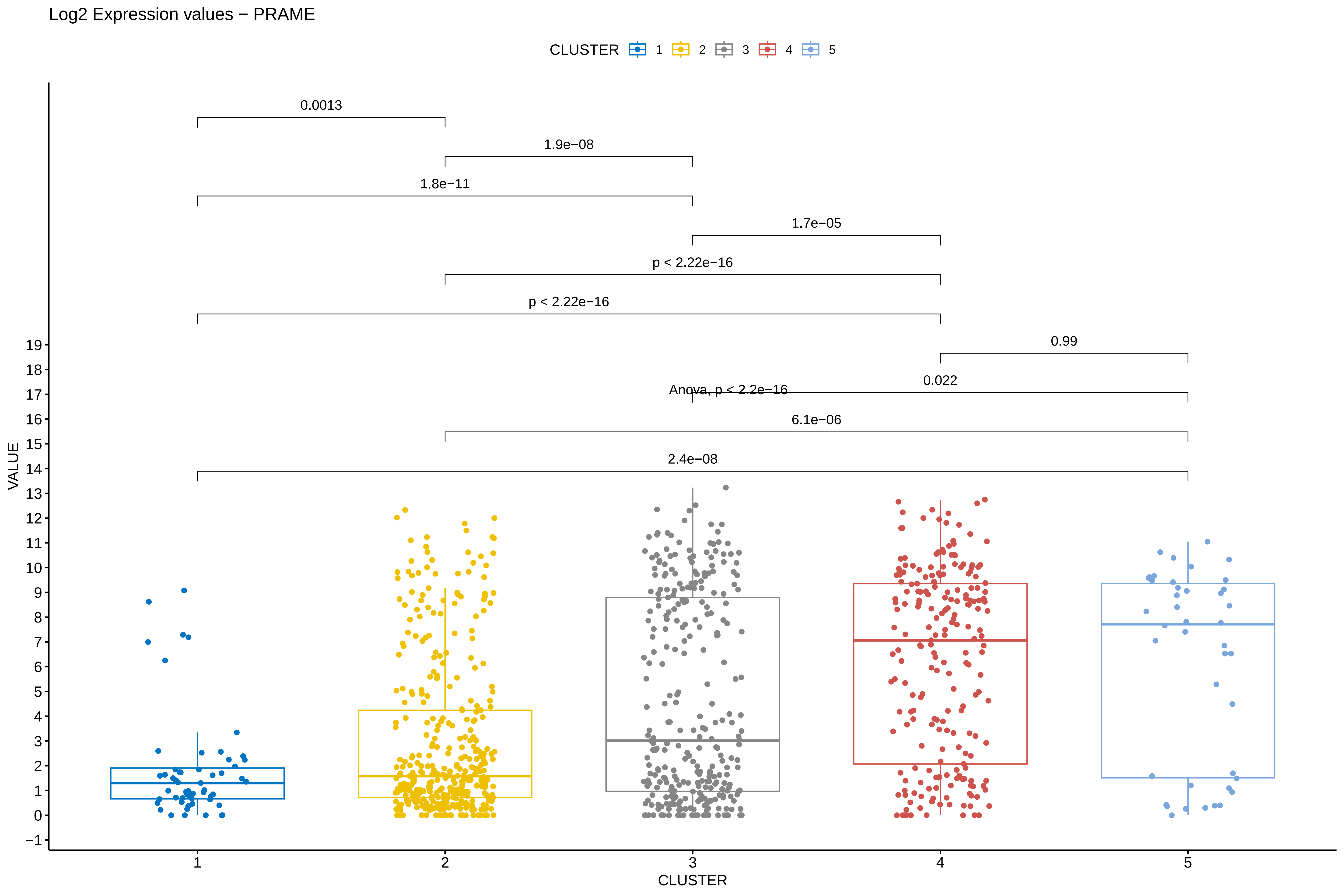
Log2 Expression values − PRAME
CLUSTER 1 2 3 4 5
0.0013
1.9e−08
1.8e−11
1.7e−05
p < 2.22e−16
p < 2.22e−16
0.99
0.022
Anova, p < 2.2e−16
6.1e−06
2.4e−08
VALUE
19
18
17
16
15
14
13
12
11
10
9
8
7
6
5
4
3
2
1
0
−1
1
2
3
4
5
CLUSTER

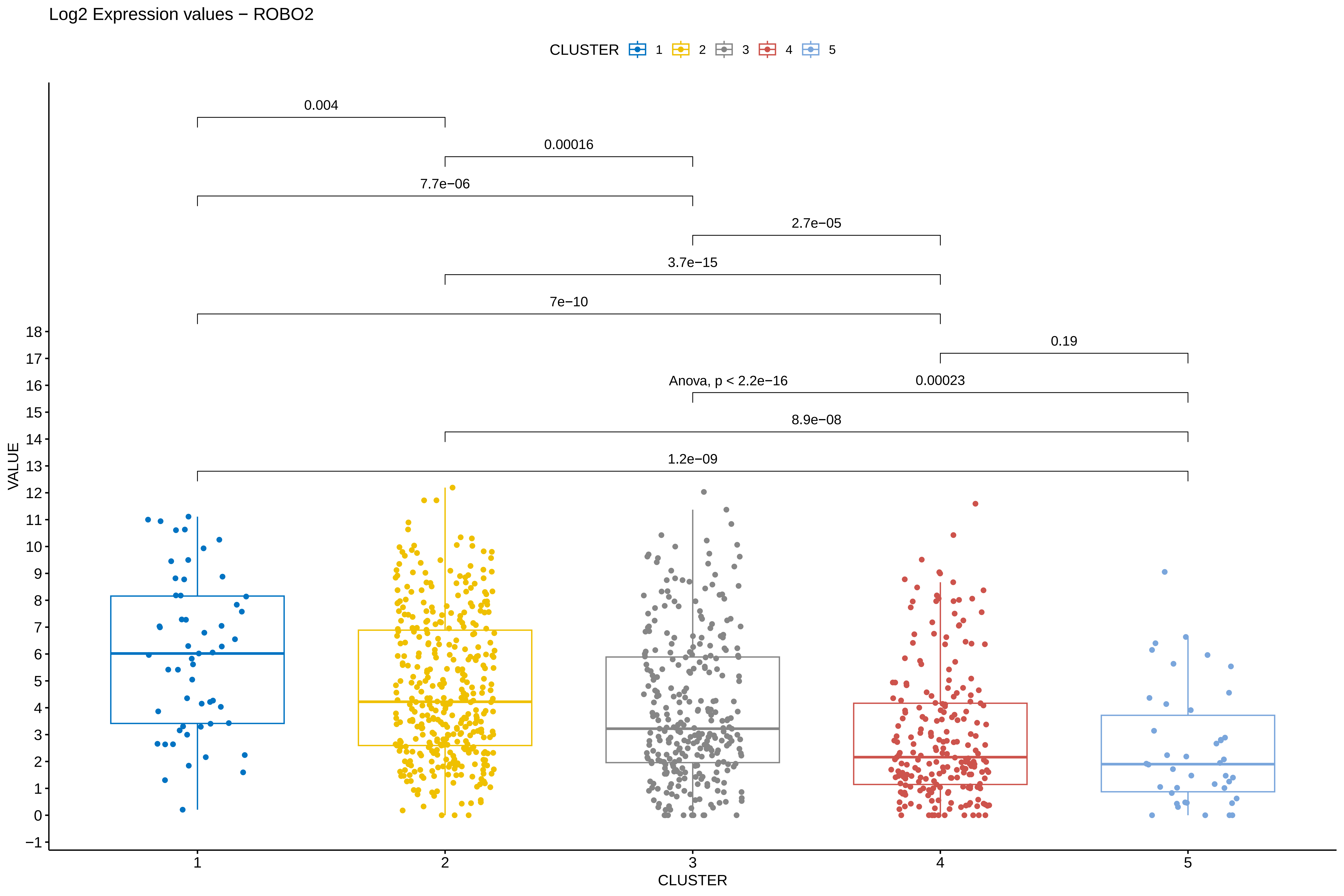
Log2 Expression values − ROBO2
CLUSTER 1 2 3 4 5
0.004
0.00016
7.7e−06
2.7e−05
3.7e−15
7e−10
0.19
Anova, p < 2.2e−16
0.00023
8.9e−08
1.2e−09
VALUE
18
17
16
15
14
13
12
11
10
9
8
7
6
5
4
3
2
1
0
−1
1
2
3
4
5
CLUSTER

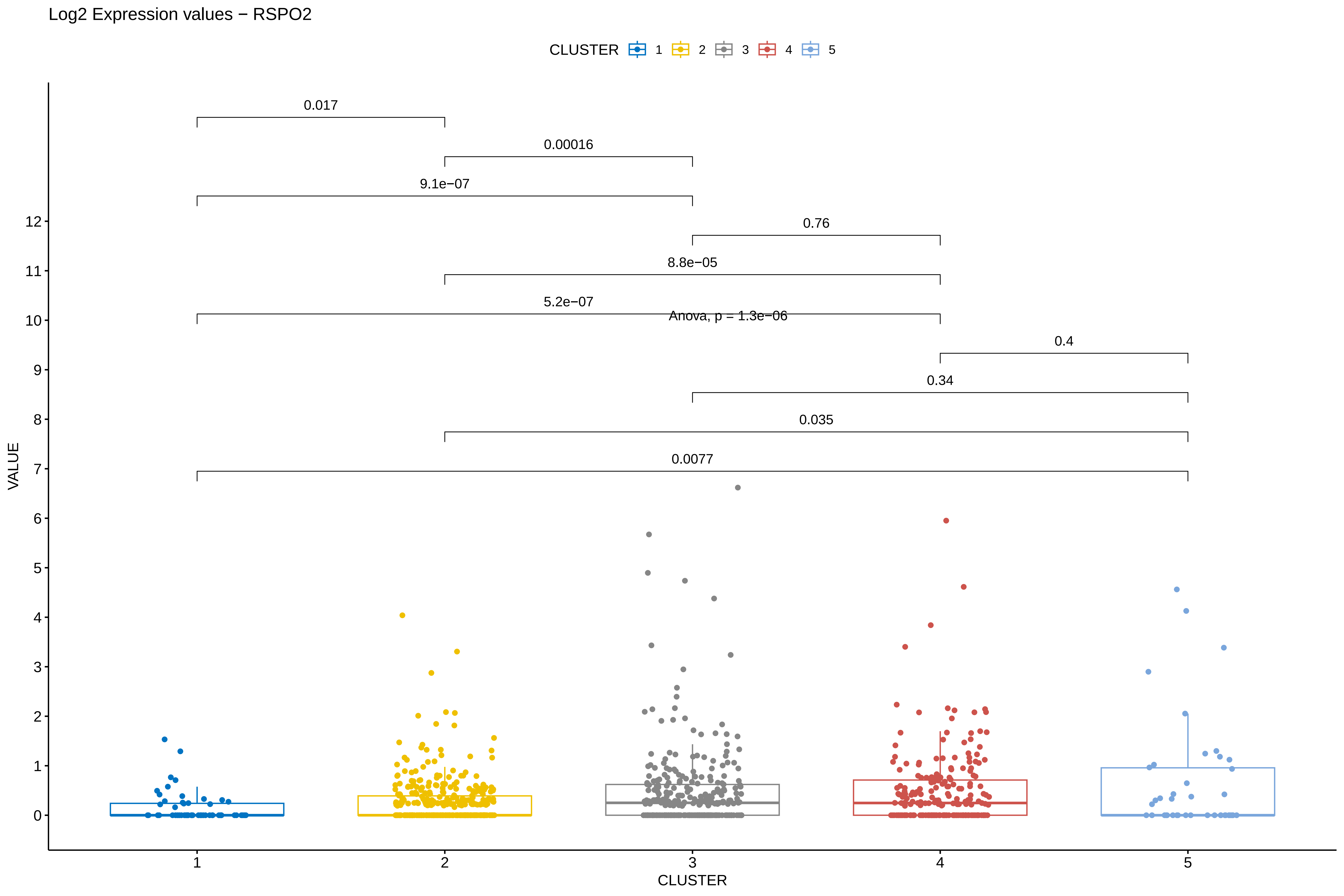
Log2 Expression values − RSPO2
CLUSTER 1 2 3 4 5
0.017
0.00016
9.1e−07
0.76
8.8e−05
5.2e−07
Anova, p = 1.3e−06
0.4
0.34
0.035
0.0077
VALUE
0 1 2 3 4 5 6 7 8 9 10 11 12
CLUSTER
1 2 3 4 5

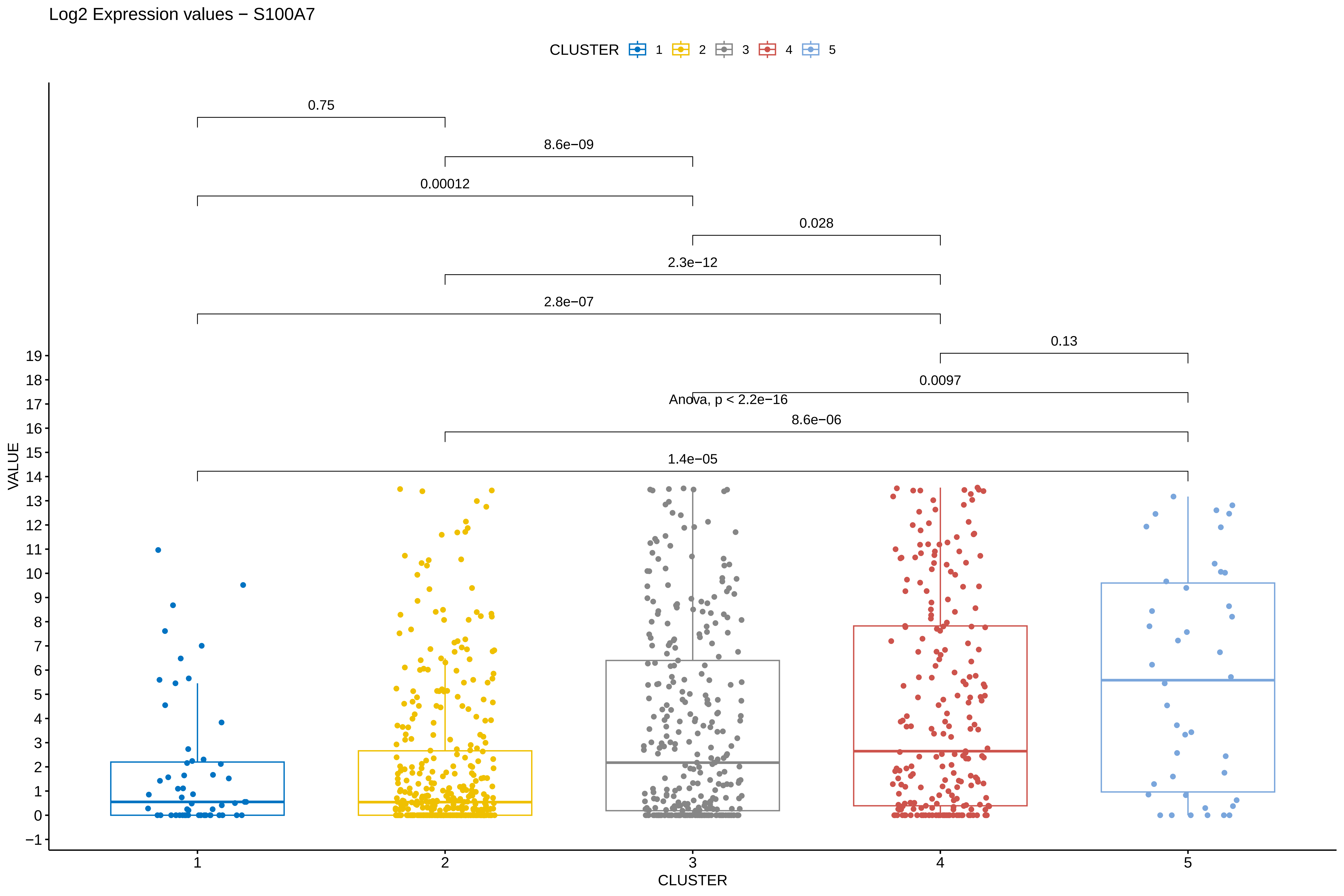

Log2 Expression values − S100A7
CLUSTER 1 2 3 4 5
0.75
8.6e−09
0.00012
0.028
2.3e−12
2.8e−07
0.13
0.0097
Anova, p < 2.2e−16
8.6e−06
1.4e−05
VALUE
19
18
17
16
15
14
13
12
11
10
9
8
7
6
5
4
3
2
1
0
−1
1
2
3
4
5
CLUSTER

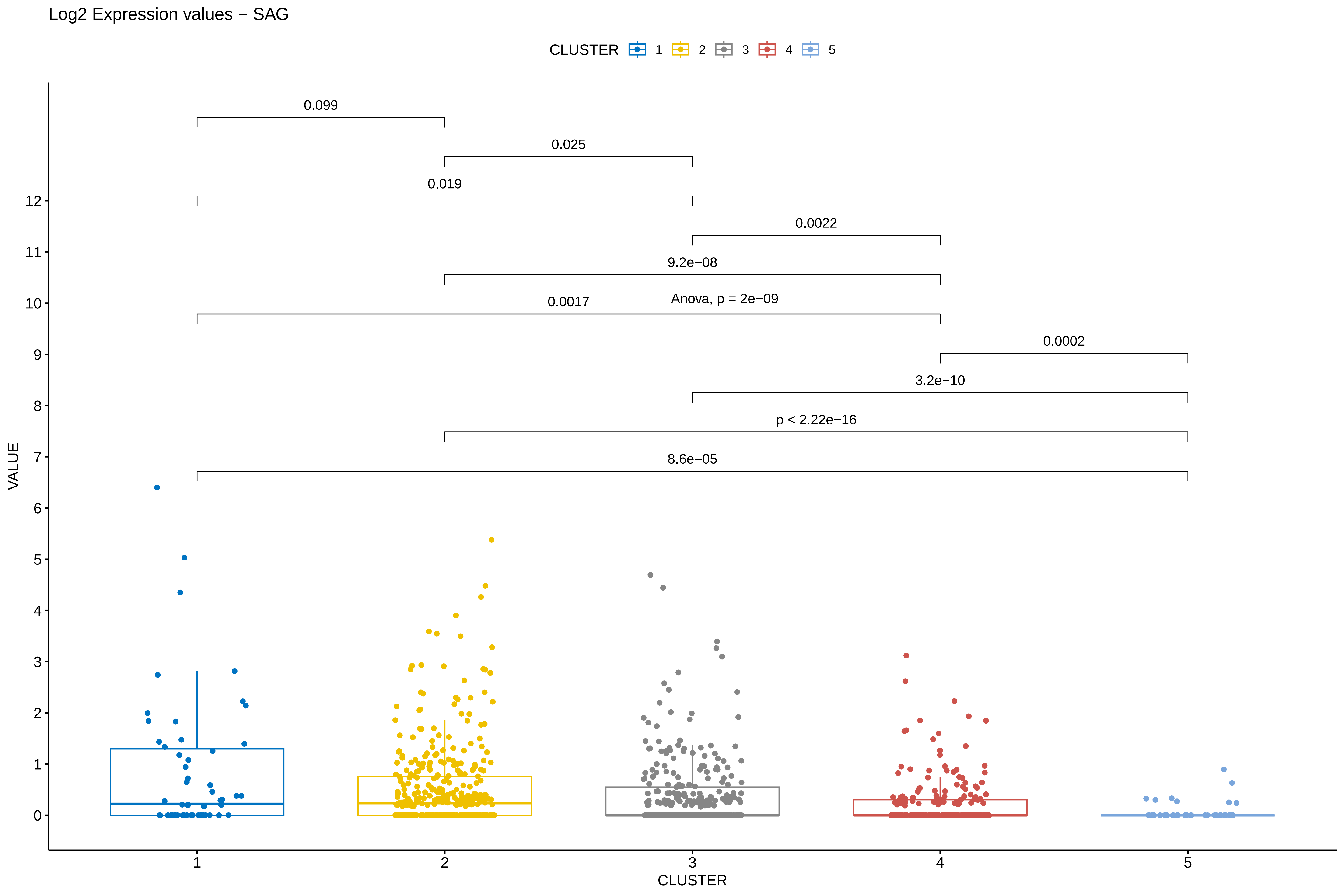

Log2 Expression values − SAG
CLUSTER 1 2 3 4 5
0.099
0.025
0.019
0.0022
9.2e−08
0.0017
Anova, p = 2e−09
0.0002
3.2e−10
p < 2.22e−16
8.6e−05
VALUE
0
1
2
3
4
5
6
7
8
9
10
11
12
1
2
3
4
5
CLUSTER

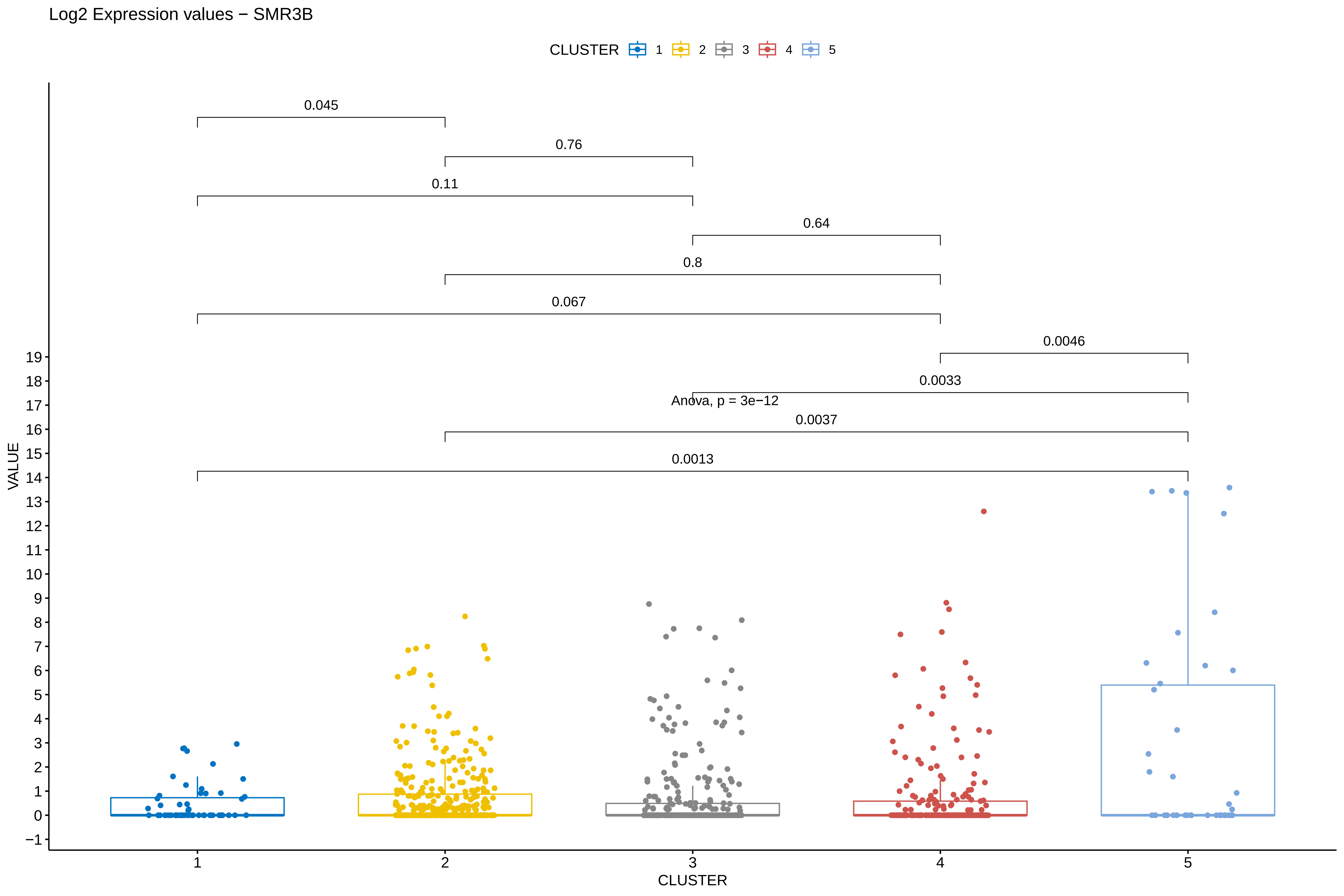
Log2 Expression values − SMR3B
CLUSTER 1 2 3 4 5
0.045
0.76
0.11
0.64
0.8
0.067
0.0046
0.0033
Anova, p = 3e−12
0.0037
0.0013
VALUE
CLUSTER
19
18
17
16
15
14
13
12
11
10
9
8
7
6
5
4
3
2
1
0
−1
1
2
3
4
5

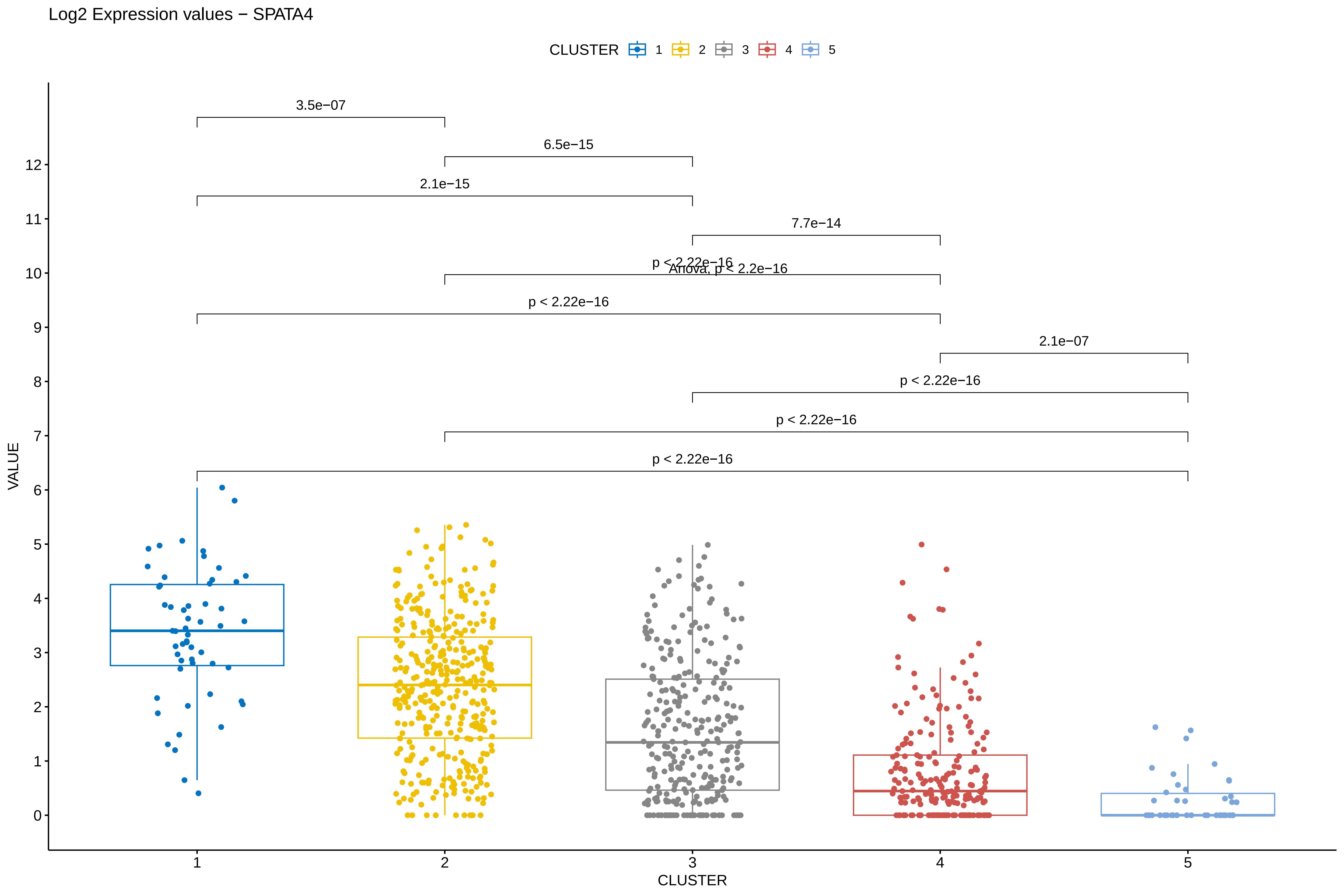
Log2 Expression values − SPATA4
CLUSTER 1 2 3 4 5
3.5e−07
6.5e−15
2.1e−15
7.7e−14
p < 2.22e−16
Anova, p < 2.2e−16
p < 2.22e−16
2.1e−07
p < 2.22e−16
p < 2.22e−16
p < 2.22e−16
VALUE
0
1
2
3
4
5
6
7
8
9
10
11
12
1
2
3
4
5
CLUSTER

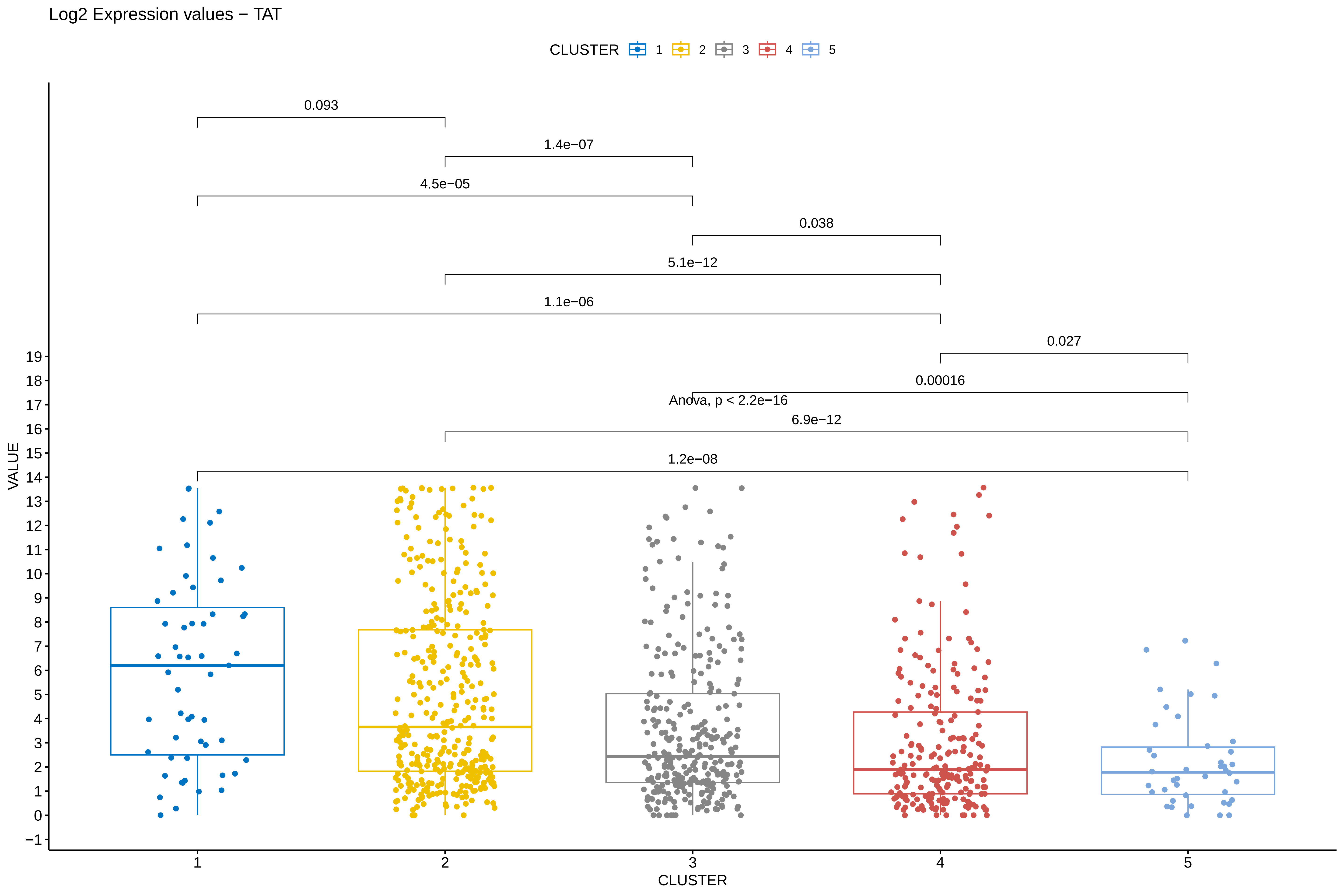
Log2 Expression values − TAT
CLUSTER 1 2 3 4 5
0.093
1.4e−07
4.5e−05
0.038
5.1e−12
1.1e−06
0.027
0.00016
Anova, p < 2.2e−16
6.9e−12
1.2e−08
VALUE
CLUSTER
19
18
17
16
15
14
13
12
11
10
9
8
7
6
5
4
3
2
1
0
−1
1
2
3
4
5

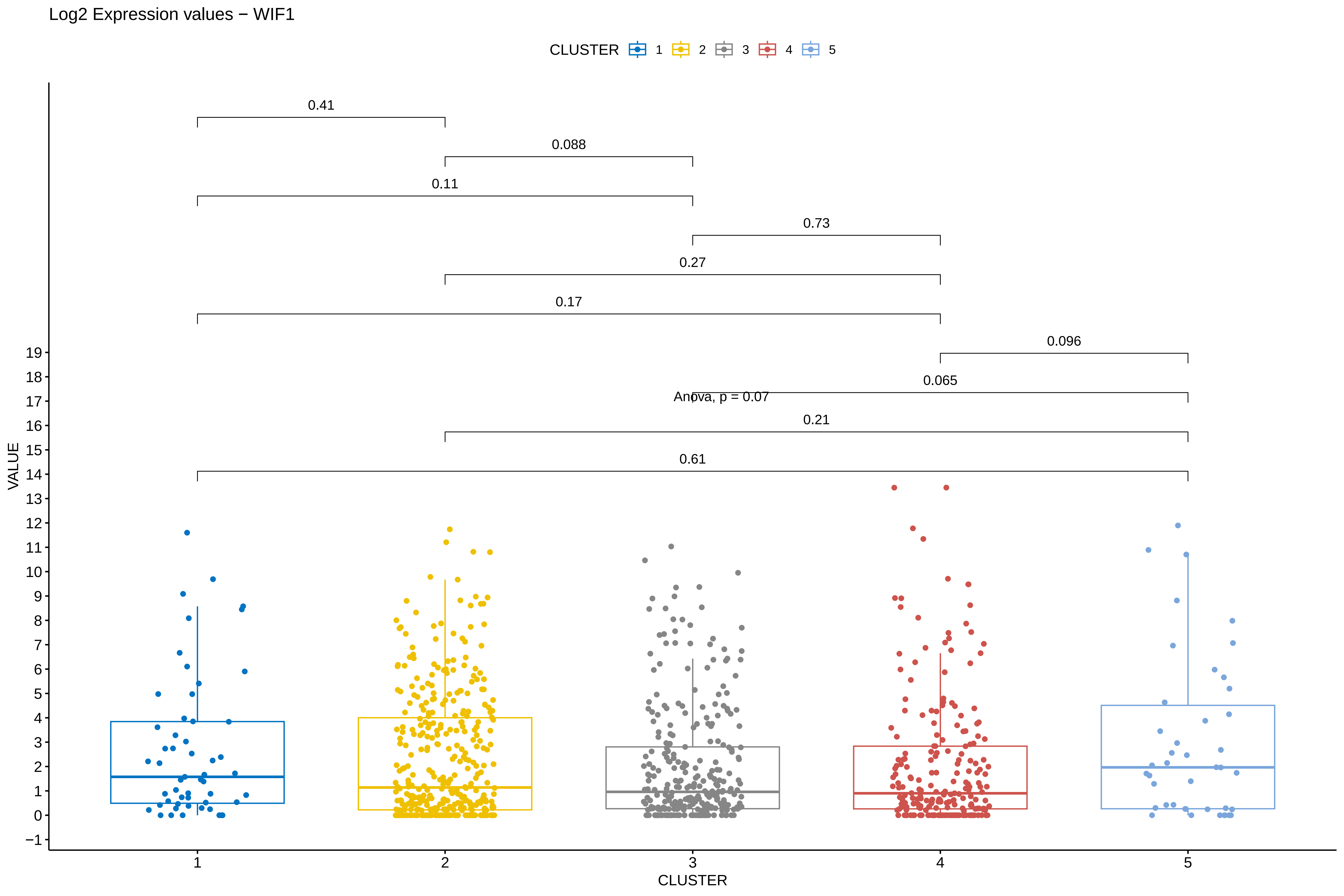

Log2 Expression values − WIF1
CLUSTER 1 2 3 4 5
0.41
0.088
0.11
0.73
0.27
0.17
0.096
0.065
Anova, p = 0.07
0.21
0.61
VALUE
19
18
17
16
15
14
13
12
11
10
9
8
7
6
5
4
3
2
1
0
−1
1
2
3
4
5
CLUSTER

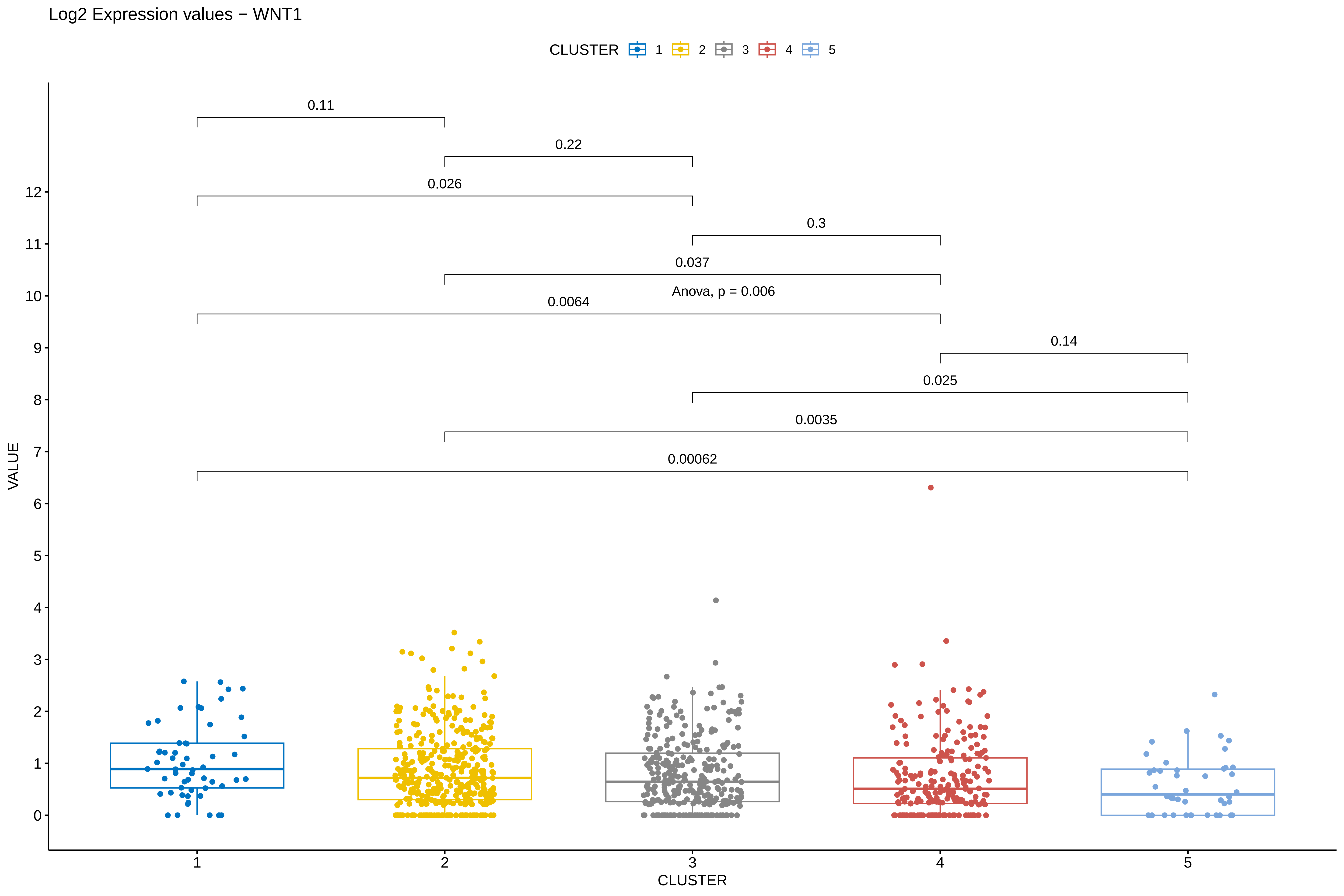

Log2 Expression values − WNT1
CLUSTER 1 2 3 4 5
0.11
0.22
0.026
0.3
0.037
Anova, p = 0.006
0.0064
0.14
0.025
0.0035
0.00062
VALUE
0
1
2
3
4
5
6
7
8
9
10
11
12
1
2
3
4
5
CLUSTER
