# Supplementary material for: Prognostic Biomarkers in Breast Cancer via Multi-Omics Clustering Analysis
Source: Int J Mol Sci. 2025 Feb 24;26(5):1943. doi: 10.3390/ijms26051943 (PMC11900291; doi:10.3390/ijms26051943)
Supplement: Supplementary file 1 [file ijms-26-01943-s001.zip › Supp Data S2.pdf]

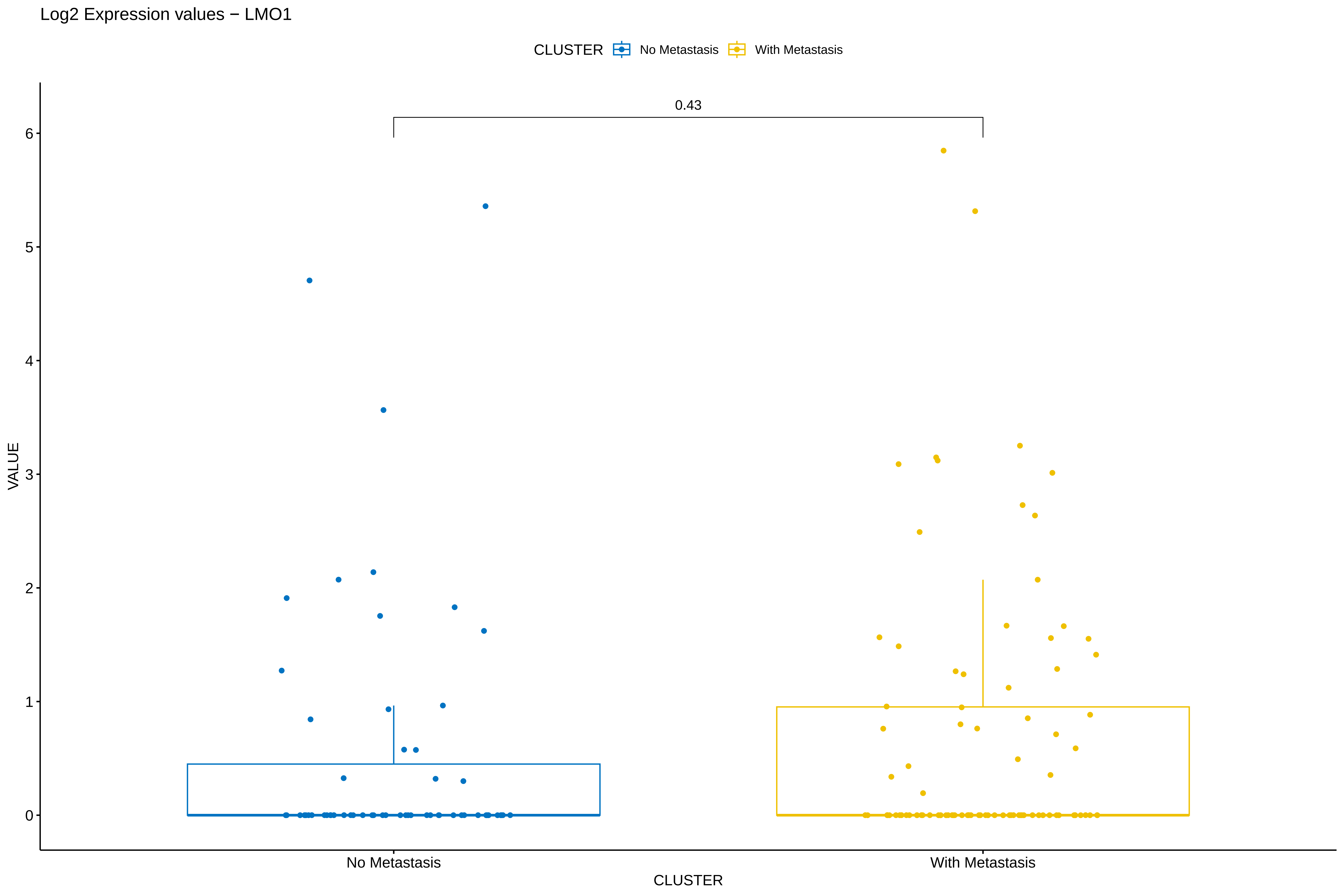

Log2 Expression values − LMO1
CLUSTER
No Metastasis
With Metastasis
0.43
VALUE
0
1
2
3
4
5
6
No Metastasis
With Metastasis
CLUSTER

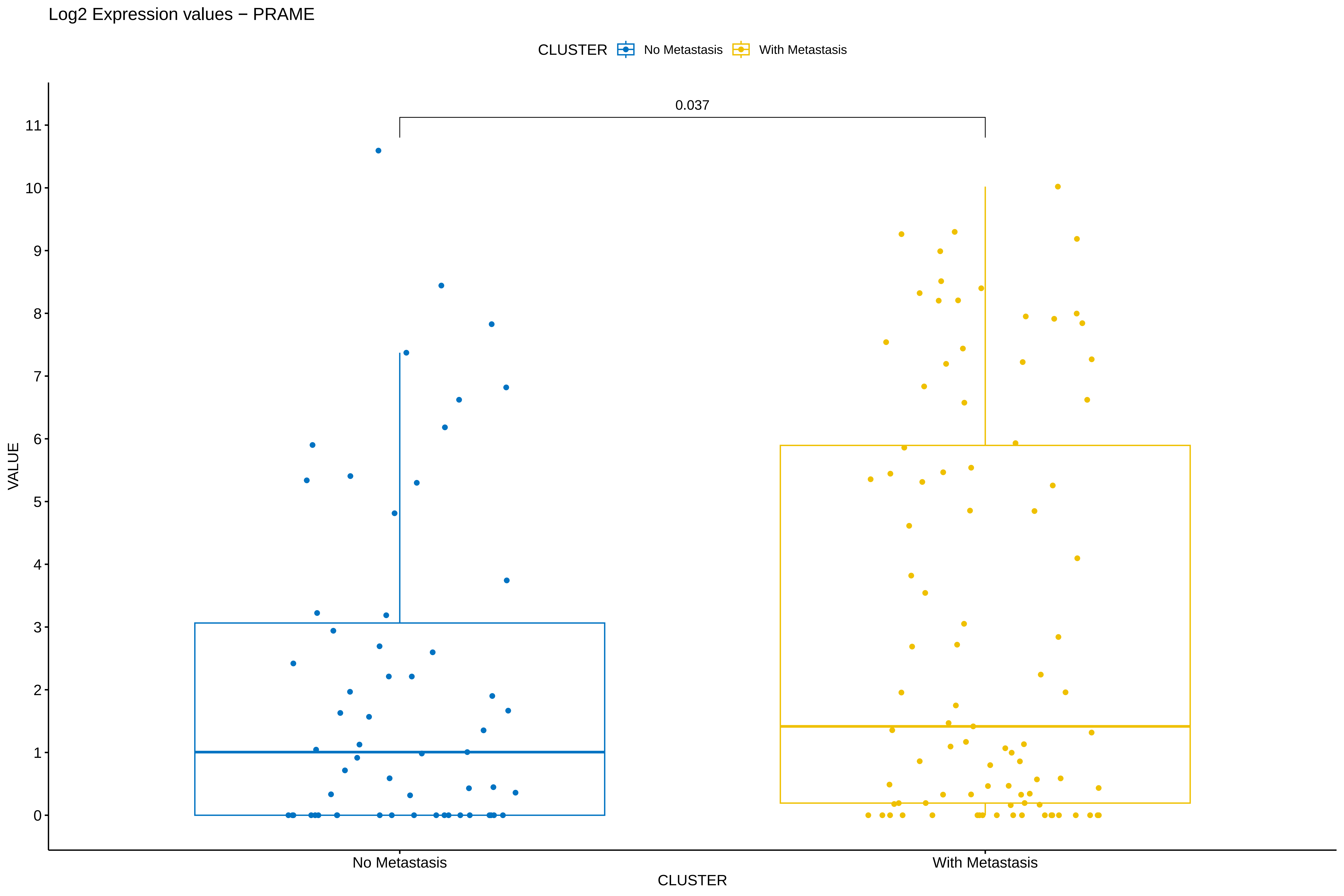

Log2 Expression values − PRAME
CLUSTER
No Metastasis
With Metastasis
0.037
VALUE
0
1
2
3
4
5
6
7
8
9
10
11
No Metastasis
With Metastasis
CLUSTER

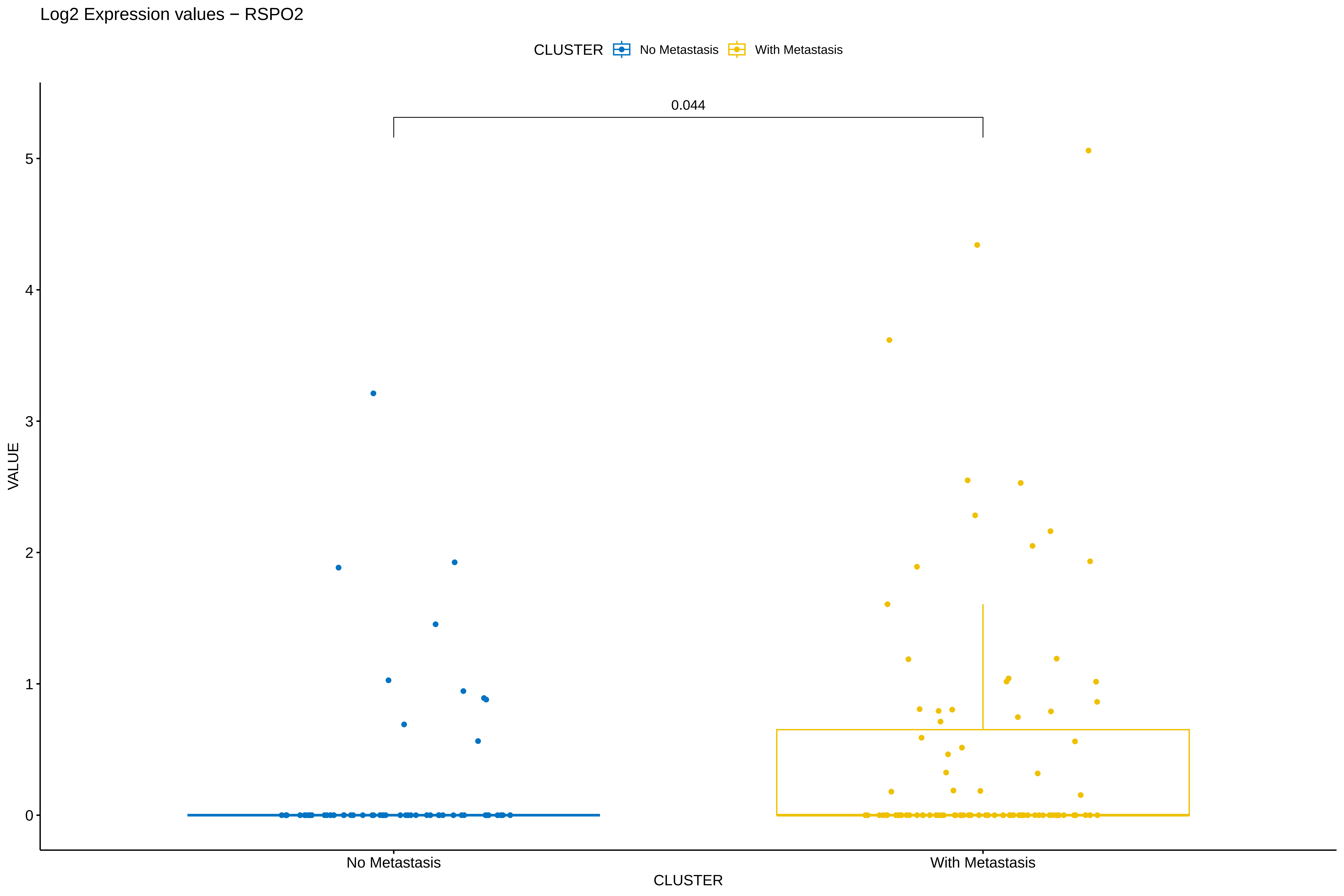

Log2 Expression values − RSPO2
CLUSTER
No Metastasis
With Metastasis
0.044
5
4
3
2
1
0
VALUE
No Metastasis
With Metastasis
CLUSTER
