# Supplementary material for: Prognostic Biomarkers in Breast Cancer via Multi-Omics Clustering Analysis
Source: Int J Mol Sci. 2025 Feb 24;26(5):1943. doi: 10.3390/ijms26051943 (PMC11900291; doi:10.3390/ijms26051943)
Supplement: Supplementary file 1 [file ijms-26-01943-s001.zip › Supp Table S1.pdf]

Significant Biomarkers in Breast Cancer $p < 0.05$	
Upregulated	Downregulated
AMH	AFF3
LMO1	CADM2
MAGEA11	CYP24A1
PF4	GPM6A
PRAME	HEPACAM
RSPO2	IFNG
S100A7	IGFALS
SMR3B	MT1M
	ROBO2
	SAG
	SPATA4
	TAT

Table S1. Significant Biomarkers in Breast Cancer p <0.05.
