# Supplementary material for: Prognostic Biomarkers in Breast Cancer via Multi-Omics Clustering Analysis
Source: Int J Mol Sci. 2025 Feb 24;26(5):1943. doi: 10.3390/ijms26051943 (PMC11900291; doi:10.3390/ijms26051943)
Supplement: Supplementary file 1 [file ijms-26-01943-s001.zip › Supplementary Figure S1.pdf]

# Workflow

Clustering based on CIMLR (multi-omics)

⬇

Enrichment of the different clusters

⬇

Differential features (not associated to survival)

⬇

Clustering based on overall survival

⬇

List of candidate biomarkers with regularized Cox regression

⬇

Analyses
