# Supplementary figures and images for: Prognostic Biomarkers in Breast Cancer via Multi-Omics Clustering Analysis
Source: Int J Mol Sci. 2025 Feb 24;26(5):1943. doi: 10.3390/ijms26051943 (PMC11900291; doi:10.3390/ijms26051943)

Log2 Expression values - LMO1

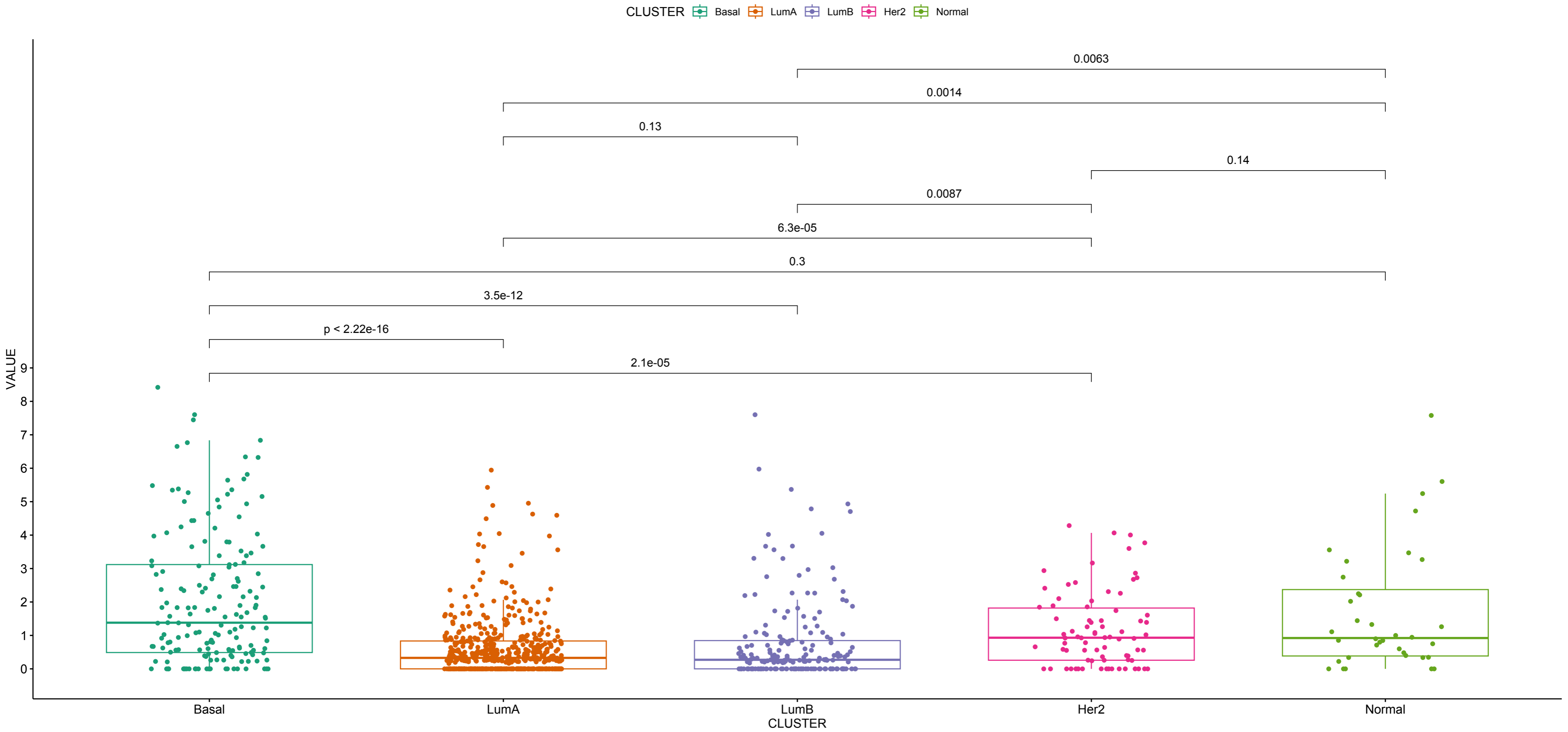

Supplement: Supplementary file 1 [file ijms-26-01943-s001.zip › Supplementary Figure S2.pdf]

Log2 Expression values - PRAME

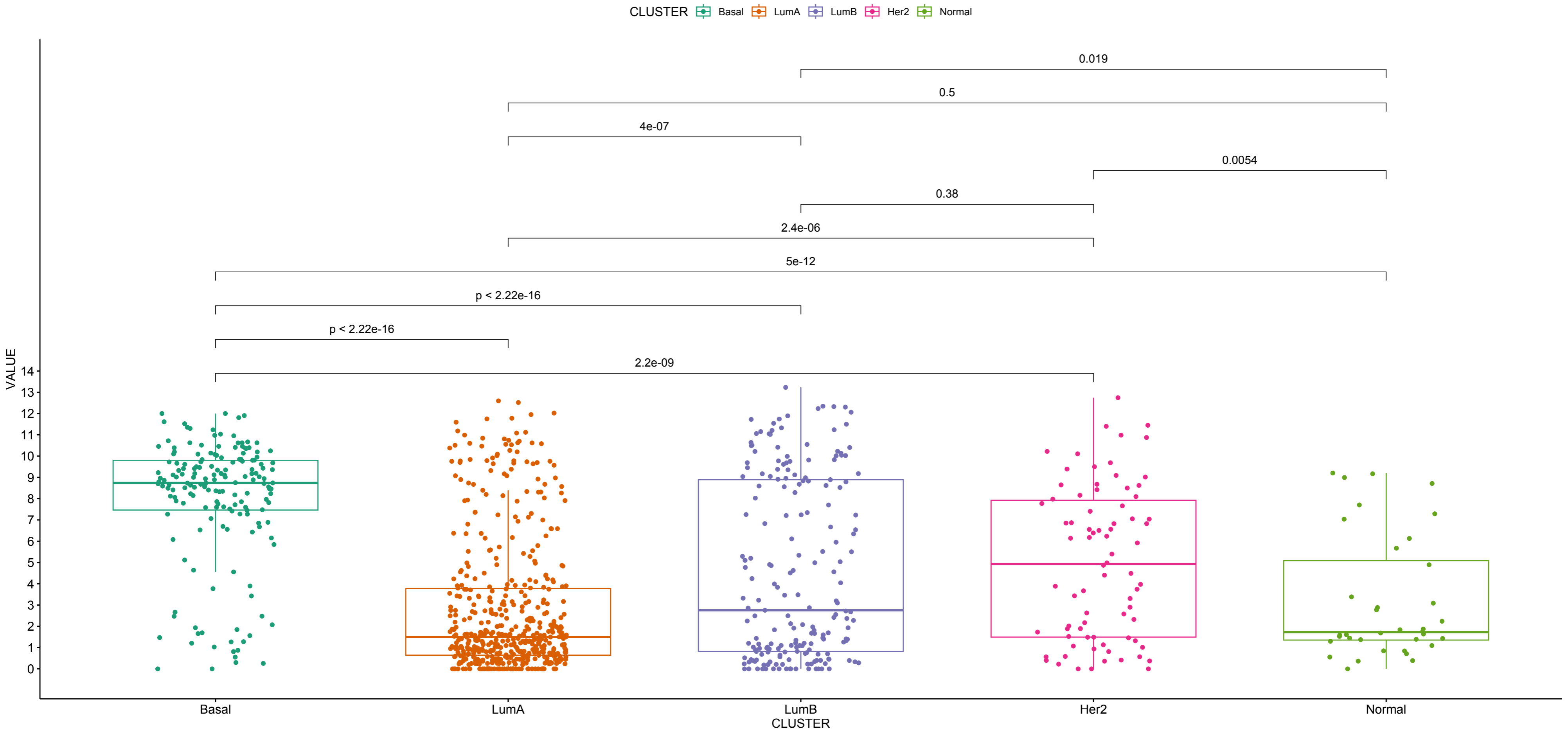

Supplement: Supplementary file 1 [file ijms-26-01943-s001.zip › Supplementary Figure S3.pdf]

Log2 Expression values - RSP02

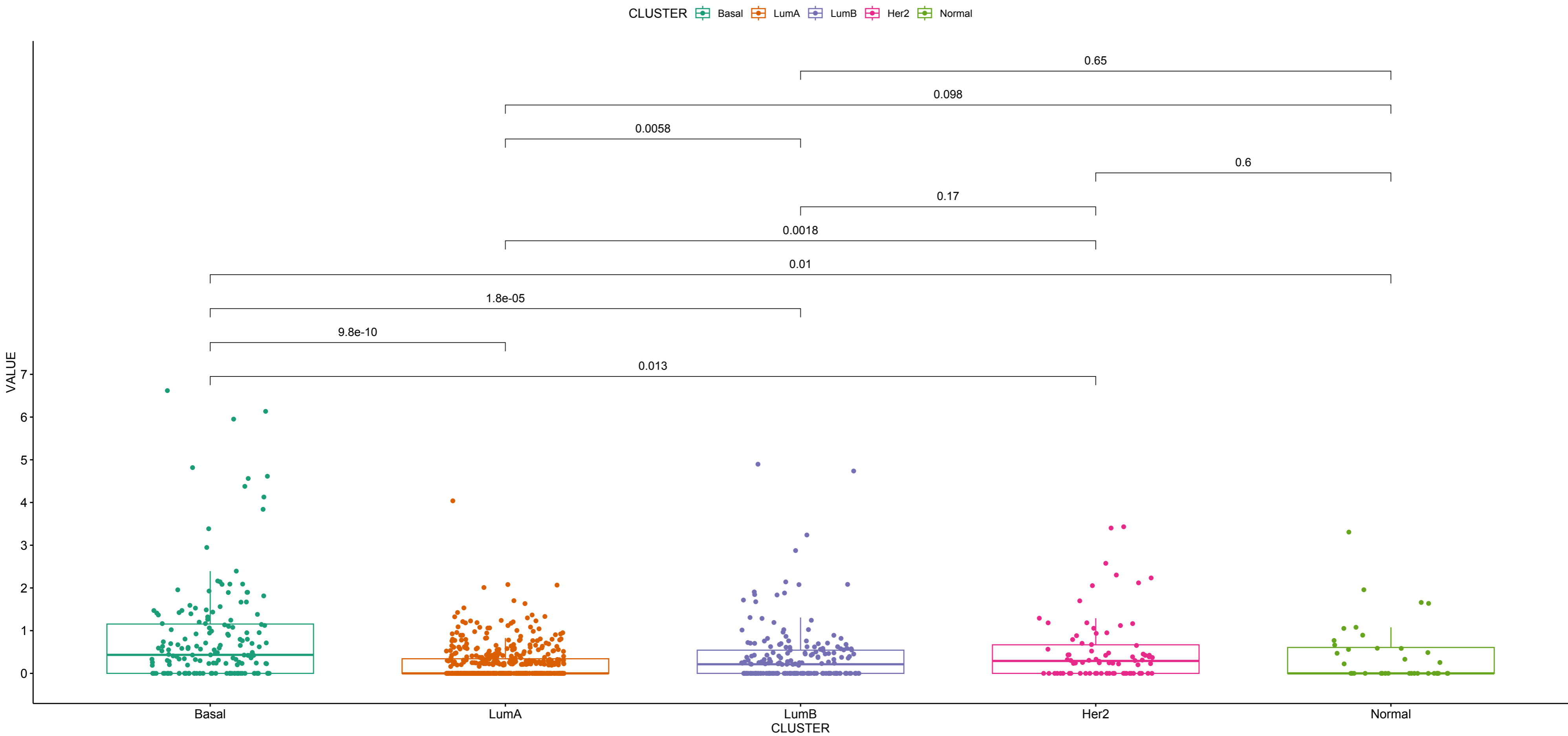

Supplement: Supplementary file 1 [file ijms-26-01943-s001.zip › Supplementary Figure S4.pdf]

# PAM50 annotations per Cluster

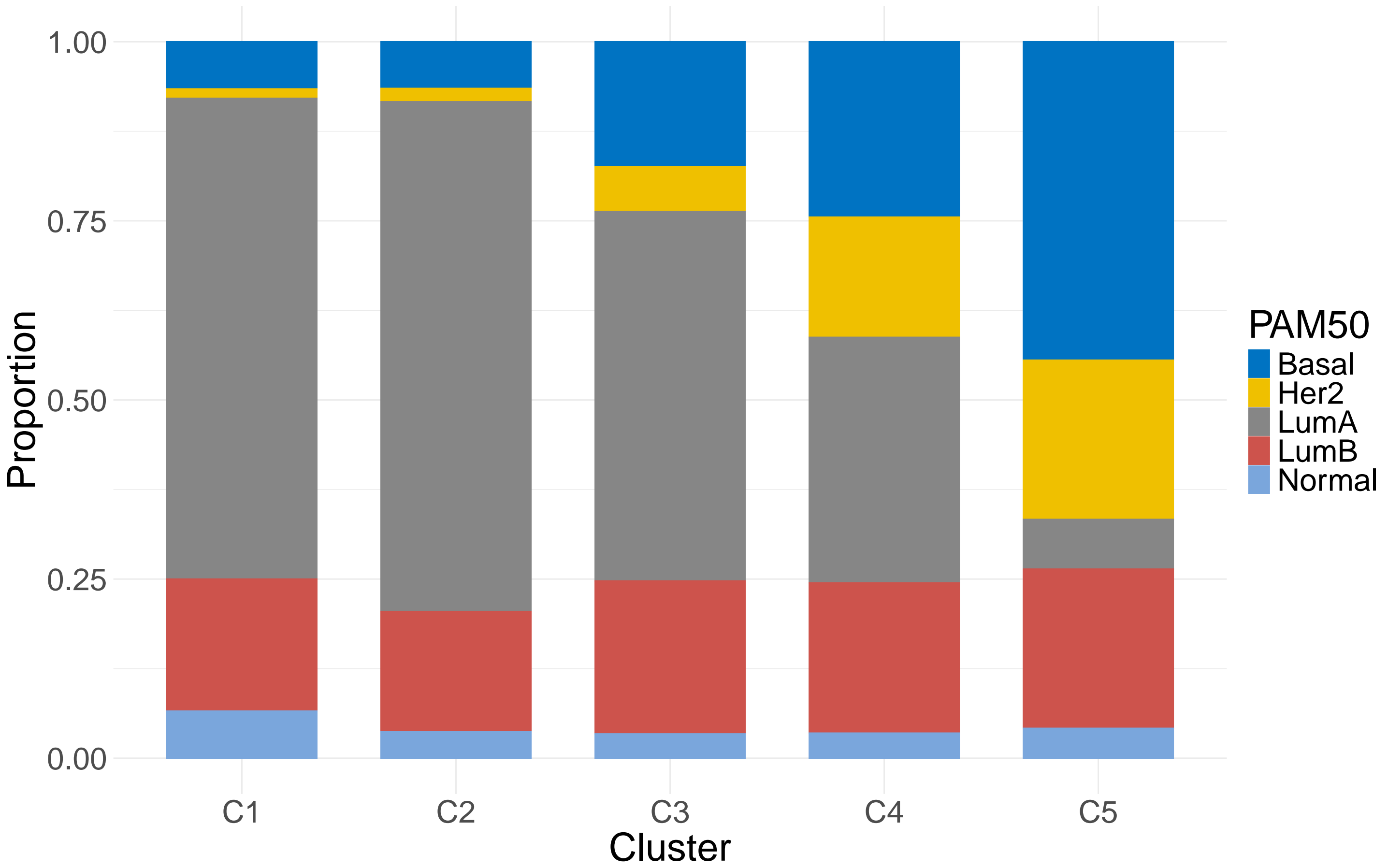

Supplement: Supplementary file 1 [file ijms-26-01943-s001.zip › Supplementary Figure S5.pdf]

a

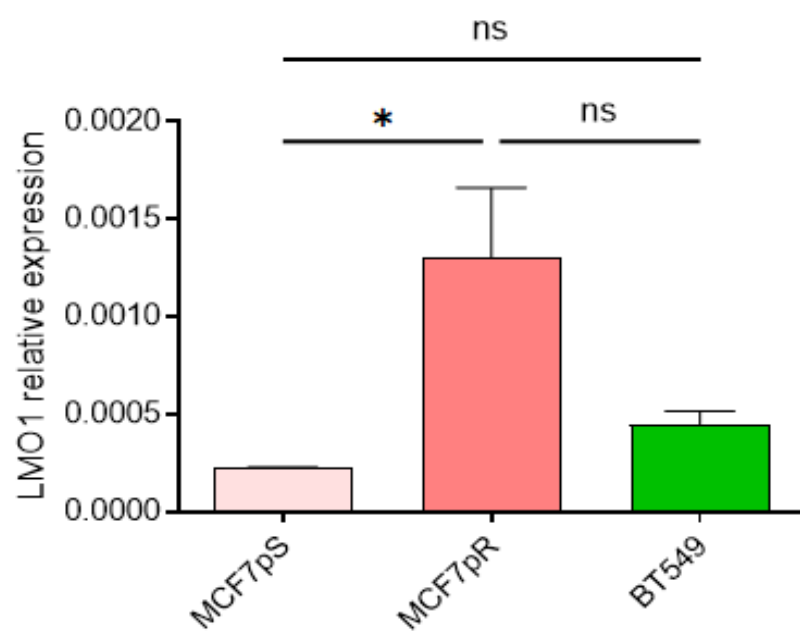

b

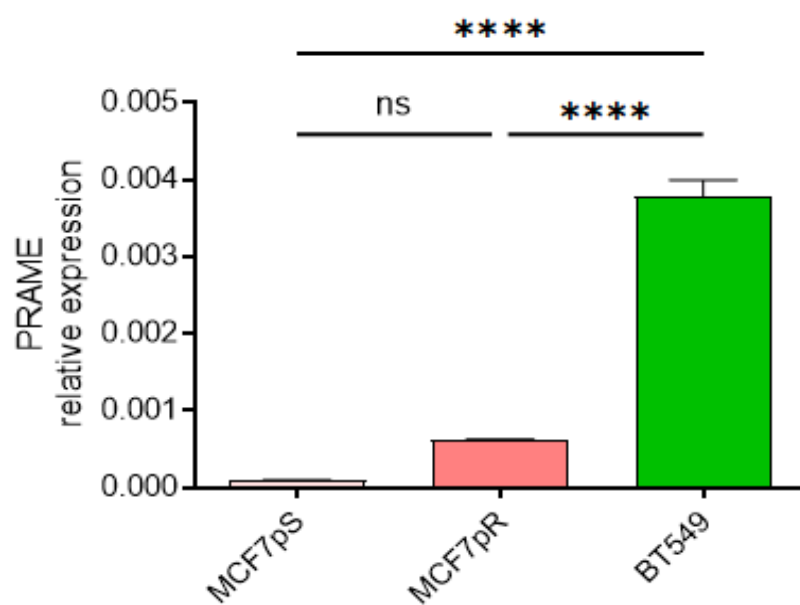

c

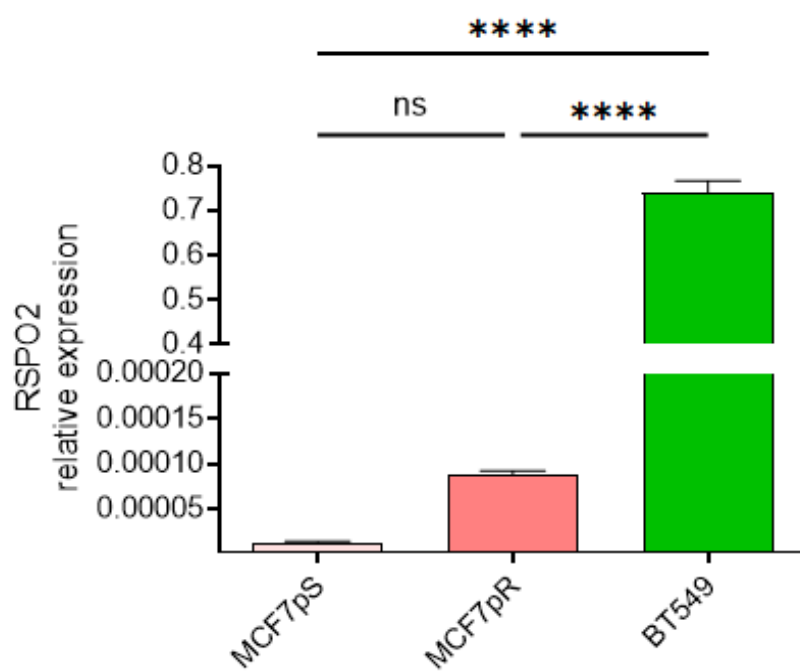

Supplement: Supplementary file 1 [file ijms-26-01943-s001.zip › Supplementary Figure S6.pdf]
